# Species-level enterosignatures predict clinical phenotypes in chronic hepatitis B and causal triangulation of gut-metabolite-CHB interactions

**DOI:** 10.3389/fmicb.2025.1683451

**Published:** 2025-10-31

**Authors:** Tingting Yuan, Jiali Chen, Jing Yang, Lianfeng Li, Shan Lu, Ji Pu, Yamin Sun, Wenchao Lin, Yubin Lu, Zhaoqin Zhu, Han Zheng, Jianguo Xu

**Affiliations:** ^1^Research Institute of Public Health, School of Medicine, Nankai University, Tianjin, China; ^2^National Key Laboratory of Intelligent Tracking and Forecasting for Infectious Diseases, National Institute for Communicable Disease Control and Prevention, Chinese Center for Disease Control and Prevention, Beijing, China; ^3^Shanghai Public Health Clinical Center, Fudan University, Shanghai, China; ^4^Institute of EcoHealth, School of Public Health, Cheeloo College of Medicine, Shandong University, Jinan, Shandong, China; ^5^National Key Laboratory of Intelligent Tracking and Forecasting for Infectious Diseases, Beijing Ditan Hospital, Capital Medical University, Beijing, China; ^6^Uniteomics Tianjin Biotechnology Co., Ltd., Tianjin, China; ^7^Research Center for Reverse Microbial Etiology, Workstation of Academician, Shanxi Medical University, Taiyuan, China

**Keywords:** chronic hepatitis B, species level, gut microbiota, enterotype, genetic causation

## Abstract

Chronic hepatitis B (CHB) remains a significant global health challenge, with research indicating the gut microbiota’s influence on disease progression, although investigations have primarily been limited to the genus level. This study conducted species-level research using the Human Gut Microbiome Analysis Database (HGMAD) to examine differences in gut microbiota between CHB patients and healthy controls (HC), to investigate enterotype associations with CHB, to assess the predictive capacity of enterosignatures for CHB phenotypes, and to determine causal relationships among gut microbiota, metabolites, and CHB. The cross-sectional investigation included 129 CHB patients and 58 HC, with fecal samples analyzed by 16S rRNA gene sequencing of the V3–V4 region. Significant differences in α-diversity and β-diversity (*P* < 0.05) were observed between the CHB and HC groups. Taxonomic analysis revealed that the high prevalent bacteria group was lower in CHB patients (61.15%) than in HC (98.05%), indicating increased gut microbiota heterogeneity in CHB. Among known bacterial species, pathogens showed higher prevalence in CHB patients (22.80% vs. 11.49%), with several potential enteropathogenic bacteria (e.g., *Bacteroides fragilis* and *Haemophilus parainfluenzae*) enriched in CHB. Dimensionality reduction and clustering analysis of gut microbiota in CHB patients revealed two distinct enterotypes: ET-P dominated by *Prevotella* and ET-B dominated by *Bacteroides*. ET-P demonstrated a correlation with elevated levels of hepatitis B virus (HBV) DNA, hepatitis B surface antigen (HBsAg), hepatitis B e antigen (HBeAg), CD4^+^T-cell count and CD8^+^T-cell count, and alpha-fetoprotein (AFP). The enterosignatures of ET-P and ET-B effectively predicted key clinical indicators: the area under the curve (AUC) was 0.78 (95% confidence interval [CI]: 0.69–0.86) for HBeAg levels, 0.86 (95% CI: 0.79–0.93) for HBV DNA levels, 0.75 (95% CI: 0.65–0.84) for AFP status, and 0.85 (95% CI: 0.77–0.92) for CD4^+^T-cell count status. Mendelian randomization (MR) analysis, integrating two gut-microbiota databases, provided genetic evidence for causal relationships between 16 species-level gut microbes and CHB. An elevated abundance of *Prevotella copri* was associated with an increased risk of CHB (OR = 1.42, 95% CI: 1.01–2.00, *P* = 0.045). Additionally, mediation MR analyses revealed potential metabolite-mediated mechanisms underlying the role of gut microbiota in CHB. Two enterotypes were identified in CHB patients, ET-P demonstrated positive associations with HBV activity and viral load. The enterosignatures derived from both enterotypes effectively predicted key CHB clinical indicators, establishing causal links and potential underlying mechanisms between gut microbiota and CHB. These findings indicate that the gut microbiota maintains close connections to HBV infection, correlating with viral load, host immune status, and disease prognosis in hepatitis B.

## Introduction

Hepatitis B virus infection presents a significant global public health challenge. Chronic hepatitis B (CHB) is characterized by infection persisting beyond 6 months (Ott et al., 2012). The persistence of covalently closed circular DNA in hepatocytes, even in cases with apparent serological resolution, maintains lifelong risks of cirrhosis and hepatocellular carcinoma (Wong et al., 2013), highlighting the critical need for innovative therapeutic approaches aligned with clinical practice guidelines from the European Association for the Study of the Liver that emphasize viral suppression (Terrault et al., 2016). The liver and gut, originating from identical embryonic germ layers, share substantial anatomical and functional connections, termed the “gut-liver axis” (Jeng et al., 2023). Mounting evidence demonstrates the vital role of gut microbiota in liver disease development, progression, and treatment response, suggesting that gut microbial translocation across the intestinal barrier contributes to inflammation and disease progression in CHB (Liu et al., 2019; Chua et al., 2023; Deng et al., 2023). However, previous studies relied on genus-level analysis and neglected gut microbiota heterogeneity in patients under disease conditions. In 2011, Arumugam et al. (2011) introduced enterotypes as a concept for clustering and grouping gut microbes, broadly categorizing them into those dominated by *Bacteroides*, *Prevotella*, and *Ruminococcus*. Various clustering approaches have yielded heterogeneous results, with increasing evidence linking enterotypes to disease progression and phenotype characteristics in conditions such as COVID-19 (Su et al., 2024), asthma (Sohn et al., 2023), inflammatory bowel disease (Caenepeel et al., 2024), and human immunodeficiency virus (HIV)/AIDS (Noguera-Julian et al., 2016). Enterotypes demonstrate stability independent of age, sex, cultural background, or geographical location (Arumugam et al., 2011), and remain unaffected by clinical characteristics or antibiotic types (Vallet et al., 2023). This stability offers an optimal approach for studying gut microbiota variations in heterogeneous populations, potentially informing therapeutic strategies and explaining disease progression patterns across clinical phenotypes (Costea et al., 2017). Recognizing that correlation does not imply causation, the causal relationship between gut microbiota and CHB, including underlying mechanisms, remains unclear. To address this, Mendelian randomization research follows the principle that “parental alleles are randomly allocated to offspring” (Davey Smith and Ebrahim, 2003). Since genotype influences phenotype, it serves as an instrumental variable to establish causal links between CHB and gut microbiota through phenotypic disease expression.

To overcome existing study limitations–including reliance on genus-level analyses, insufficient consideration of inter-individual patient heterogeneity, and inability to establish causal relationships between gut microbiota and CHB–this research conducted a species-level analysis using the Human Gut Microbiome Analysis Database (Wang et al., 2025). The study aimed to examine enterotype-CHB relationships and develop predictive models for clinical phenotypes using enterotype-specific microbial signatures (Enterosignatures) (Frioux et al., 2023). The methodology integrated clinical disease phenotype indicators, gut microbiome profiles, and single nucleotide polymorphisms identified through genome-wide association studies linked to gut microbiota, metabolites, and CHB. Through defining CHB patient enterotypes and identifying phenotype-correlated enterosignatures, this research validated the causal relationship between gut microbiota and CHB.

## Materials and methods

### Study population and sample collection

This study recruited consecutive CHB patients hospitalized at the Shanghai Public Health Clinical Center. A total of 129 patients with CHB participated in the study, excluding those with HIV, hepatitis C virus, hepatitis D virus, and other viral hepatitis infections. Hepatitis B virus (HBV) DNA, hepatitis B e antigen (HBeAg), hepatitis B surface antigen (HBsAg), HBsAb, and HBeAb levels were monitored during follow-up.

The exclusion criteria encompassed: (1) HBV infection within the past 3 months; (2) antibiotic use within 3 months; (3) probiotic use within 3 months; (4) concomitant hypertension; (5) diabetes; (6) obesity or significantly low body weight; (7) obvious atherosclerosis; (8) chronic kidney disease; (9) history of gastrointestinal surgery; (10) inflammatory bowel disease; (11) irritable bowel syndrome; (12) malignant tumors; (13) autoimmune diseases; (14) Parkinson’s disease, Alzheimer’s disease, and stroke; (15) mental illness; and (16) pregnant or lactating women.

**FIGURE 1:**
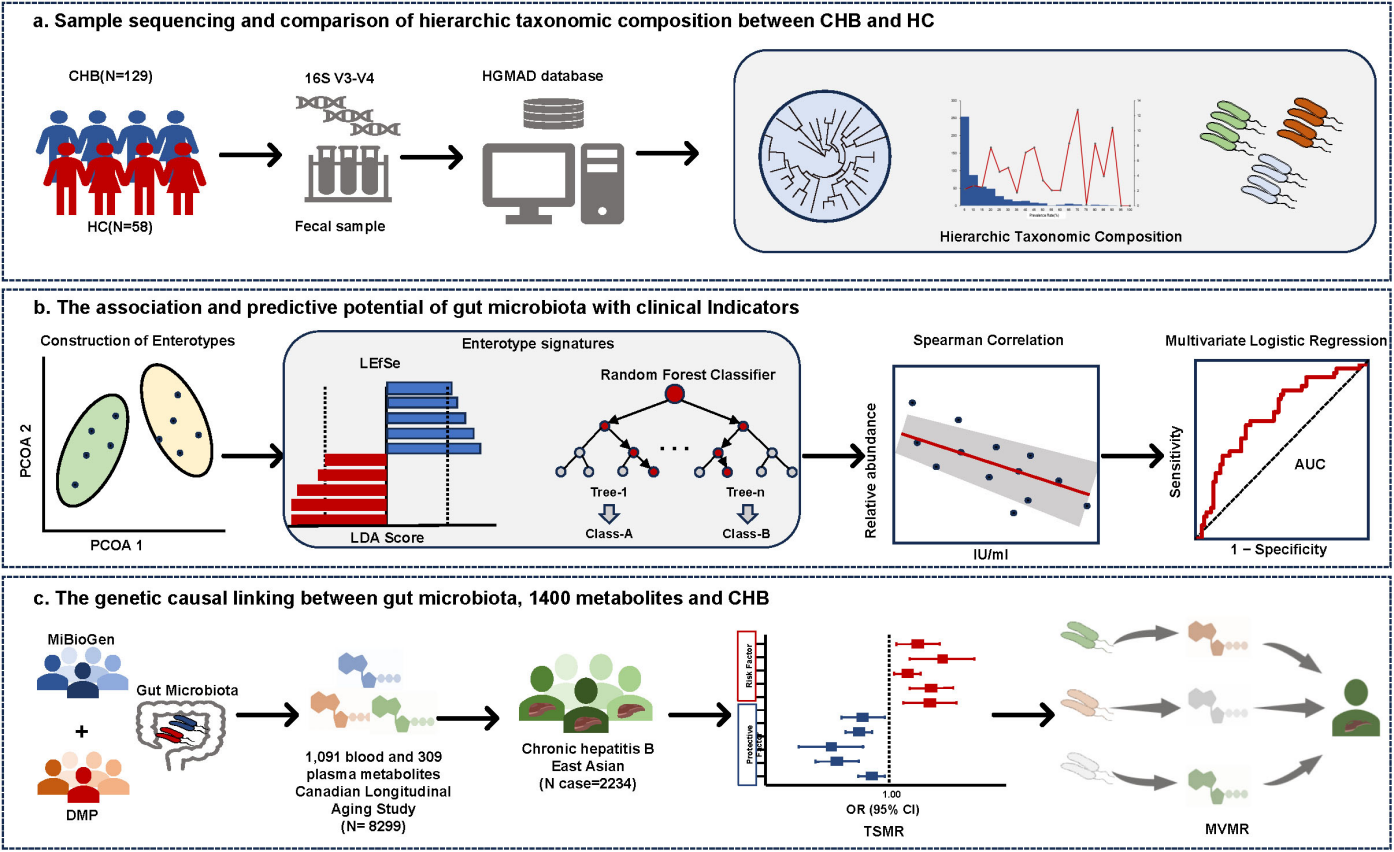
Study overview. (A) Taxonomic profiling of gut microbiota in CHB and HC. Microbial composition at the species level was characterized using 16S rRNA V3-V4 amplicon sequencing, with taxonomic annotation performed via the Human Gut Microbiome Analysis Database (HGMAD) developed by our research group. (B) Enterotype construction and microbial signature identification in CHB. Enterotypes were constructed based on gut microbiota composition. Differential taxonomic signatures between enterotypes were identified using linear discriminant analysis effect size (LEfSe) and random forest models. Spearman correlation analysis and predictive model construction were conducted to evaluate associations between enterotype-specific microbiota and clinical parameters. (C) Causal inference and mediation analysis. Two-Sample Mendelian Randomization (TSMR) and Multivariable Mendelian Randomization (MVMR) were employed to elucidate causal relationships between gut microbiota, 1,400 metabolites, and CHB, while mediation effects of metabolites on microbiota–CHB interactions were quantified.

The Ethics Committee of the Shanghai Public Health Clinical Center approved the study (approval number: 2019-S043-02). For the healthy control (HC) group, gut microbiological data from 58 healthy individuals previously published by our research group were utilized (Yang et al., 2020). These healthy individuals had no gastrointestinal symptoms (e.g., nausea, abdominal pain, diarrhea), gastrointestinal diseases (e.g., gastroenteritis, gastrointestinal infections), autoimmune diseases, or psychiatric disorders. The remaining fecal samples from these participants were collected for analysis after testing at the hospital laboratory. The study design appears in Figure 1.

### Library construction and sequencing

DNA extraction from fecal samples was performed using the QIAamp Fast DNA Stool Mini Kit (Qiagen, Germany) according to the manufacturer’s protocol. The V3–V4 region of the 16S rRNA gene was amplified from the extracted DNA using the barcoded primers 338F (5′-ACTCCTACGGGAGGCAGCAG-3′) and 806R (5′-GGACTACHVGGGTWTCTAAT-3′). Quality assessment was performed on all samples to ensure sequencing standards were met. Subsequently, all qualified samples were sequenced at Shanghai Majorbio Bio-pharm Technology Co., Ltd.

### 16S rRNA gene sequencing analysis

Paired-end reads were separated by their unique barcodes, trimmed to remove barcode and primer sequences, then merged using BBMerge (Bushnell et al., 2017) to combine overlapping fragments into raw tags. Raw tags underwent quality filtering using fastp (v0.23.1) to produce high-quality clean tags (Bokulich et al., 2013). Effective tags were processed using QIIME2 (v2022.02) with DADA2/deblur to generate amplicon sequence variants (ASVs). Taxonomic classification was performed using VSEARCH against the HGMAD database (DOI: 10.6084/m9.figshare.2728140) within the QIIME2 pipeline (Wang et al., 2025), a reference database established by our research group for species-level characterization of gut microbial communities. Alpha diversity indices, including ACE, Shannon, and Simpson diversity indices, were calculated using the diversity function of the vegan R package. For beta diversity visualization, Bray-Curtis distances between samples were computed and represented on principal coordinates analysis (PCoA) ordination plots. Permutational multivariate analysis of variance (PERMANOVA) was conducted using the adonis2 function to assess sample dispersion across indicated variables. The microeco package was utilized to evaluate gut microbiota differences, with genera identified by an effect size linear discriminant analysis (LDA) score > 2 considered differentially enriched taxa. Microbial functional pathway analysis was conducted using PICRUSt2, based on the Kyoto Encyclopedia of Genes and Genomes (KEGG) and Clusters of Orthologous Groups (COG), following the PICRUSt2 protocol (Douglas et al., 2020).

### Classification of the gut microbiota based on enterotypes

To demonstrate compositional differences in microbial populations, samples were clustered using the partitioning around medoids (PAM) clustering algorithm, with optimal cluster numbers determined using the Calinski-Harabasz (CH) index, as previously described (Lovmar et al., 2005). Fecal microbiota samples were clustered using the Jensen–Shannon divergence at the genus level, accounting for both abundance and phylogenetic distance of shared genera (Arumugam et al., 2011; Costea et al., 2017).

### MR analyses and mediation analysis

We conducted a two-sample MR analysis to examine the causal relationship between the gut microbiota and CHB. A two-step and multivariable MR approach was employed to identify the mediating effect of metabolites on the relationship between the gut microbiota and CHB. Genetic variants influencing the relative abundance of microbial taxa were identified using microbiota quantitative trait loci (mbQTL) mapping. The analysis defined 131 genera, 35 families, 20 orders, 16 classes, and 9 phyla (Kurilshikov et al., 2021). However, this genome-wide association study (GWAS) dataset lacked species-level gut microbiota data. Therefore, we integrated data from the DMP to enhance our analysis. The GWAS summary data for DMP included 5 phyla, 10 classes, 13 orders, 26 families, 48 genera, and 105 species (Lopera-Maya et al., 2022). The GWAS data collection for 1,400 metabolites was conducted comprehensively using multiple datasets (Chen et al., 2023). The GWAS summary statistics were obtained from the EBI GWAS Catalog (accession numbers: GCST90199621 to GCST90201020), and outcome data were sourced from the GWAS Catalog database^1^ using identifier GCST90018584 for Asian populations (Sakaue et al., 2021). For instrumental variable screening, we selected single nucleotide polymorphisms (SNPs) (F statistics = β^2^/SE^2^) with genome-wide significance (*P* < 1 × 10^–5^) and F statistics ≥ 10, indicating strong correlation with exposure factors. For 1,400 metabolites, the significance level was adjusted to 5 × 10^–6^. The independence test criterion evaluated selected instrumental variables with a linkage disequilibrium threshold of r^2^ < 0.001 and a genetic distance of 10,000 kb. The inverse variance weighted (IVW) approach served as the primary analysis method. MR results were expressed as odds ratios (ORs) with 95% confidence intervals (CI). Results were considered statistically significant when IVW *P*-values were below 0.05, with multiple statistical methods used for sensitivity analysis. MR-PRESSO tests were tests were performed to detect horizontal pleiotropy (*P* < 0.05) and remove outlier SNPs (Bowden et al., 2015). The MR Egger intercept assessed pleiotropy levels (Verbanck, 2018). Cochran’s Q test evaluated heterogeneity among instrumental variables. A fixed-effects model was used for IVW calculations when *P* > 0.05. All MR analyses were performed using R (version 4.3.2; R Foundation for Statistical Computing, Vienna, Austria) with “TwoSampleMR,” “GWASTools,” “SNPRelate,” “plinkQC,” “gwasglue,” and “VariantAnnotation” packages.

### Statistical analysis

Continuous variables were presented as means ± standard deviation (SD), with statistical comparisons conducted using independent *t*-tests between two groups and one-way ANOVA for multiple groups. Non-normally distributed variables were expressed as medians and interquartile ranges (IQR), while categorical variables were described as frequencies and percentages. Non-normally distributed continuous variables were compared using the Kruskal–Wallis H test across multiple groups, with Dunn’s multiple comparisons test applied for significant results. Categorical variables were compared using the Chi square test. Correlations between variables were computed using Spearman’s correlation. Multivariable logistic regression was used to assess associations between multiple independent variables and categorical dependent variables. A two-step method was adopted to select signatures for different enterotypes. Initially, a random forest model (ntree = 500) identified the ten most predominant species as candidates for distinguishing different enterotypes. Subsequently, linear discriminant analysis effect size (LEfSe) analysis was applied to select different species, with a threshold for LDA score > 3 and *P* < 0.05 (Gao et al., 2024). Finally, overlapping species from both methods were designated as signatures. The receiver operating characteristic (ROC) curve was used to determine optimal cutoff points in diagnostic tests and predictive models, plotting true positive rates against false positive rates to reflect sensitivity–specificity trade-offs. Statistical analyses were performed using SPSS version 21.0 and visualized using R (version 4.3.2) with specified packages. The significance threshold was established at a two-sided *P*-value < 0.05.

## Results

### Hierarchic taxonomic composition of gut microbial community between hepatitis B patients and healthy individuals

**FIGURE 2:**
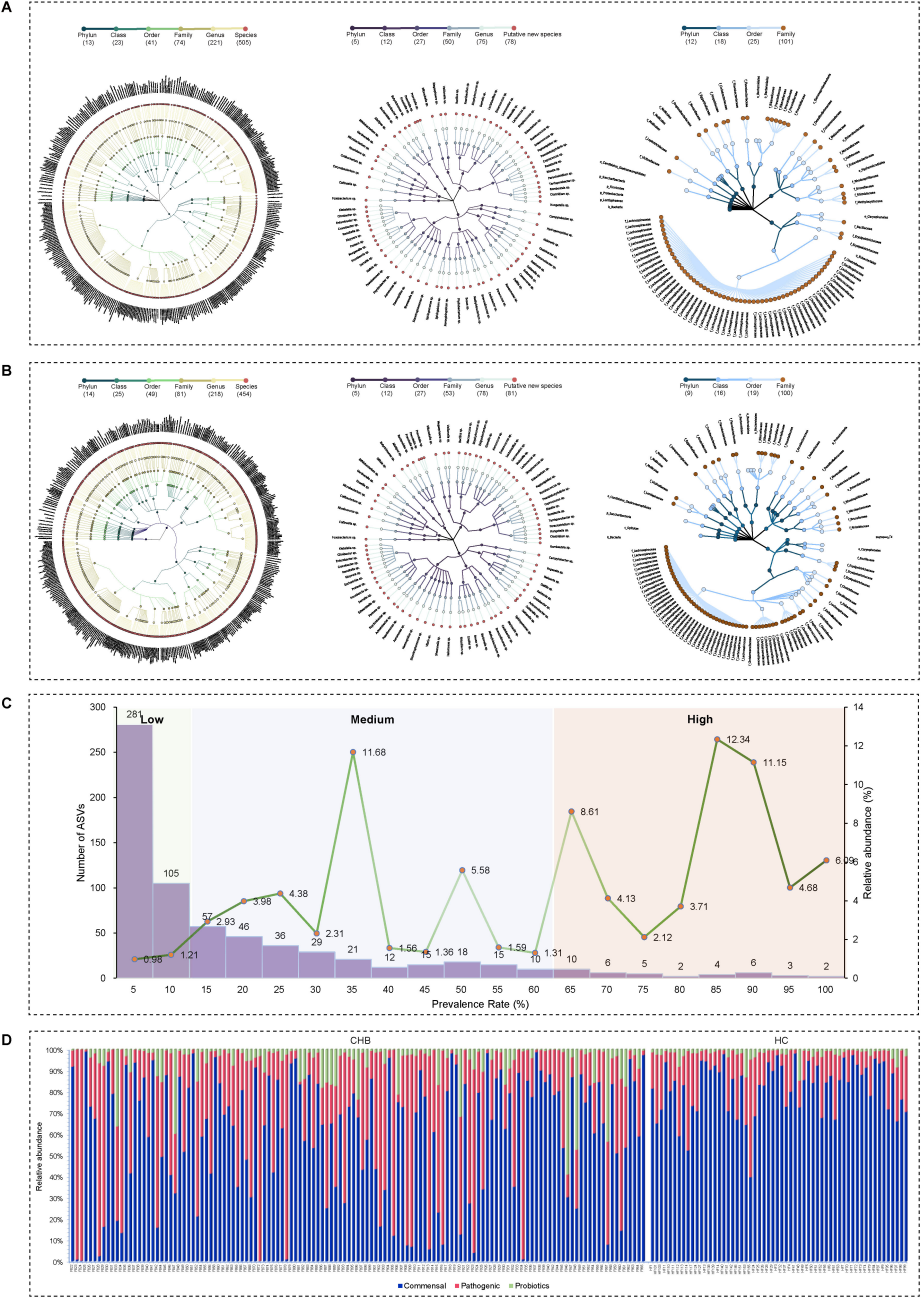
Taxonomic profiles of the gut microbial community in CHB patients and HC. (A) Taxonomic tree of CHB patients. (B) Taxonomic tree of HC. (C) Low-, medium-, and high-prevalence bacterial groups in CHB patients, showing the numbers (left axis) and relative abundance (right axis) of ASVs. (D) Relative abundance of probiotic, commensal, and potential pathogenic bacteria in CHB patients and HC.

The study cohort consisted of 129 CHB patients (90 males and 39 females) with a median age of 52 years (IQR, 40.5–58), while the 58 HC participants had a median age of 33 years (IQR, 26.5–45.5), with males comprising 48% of the group. The V3–V4 variable region of the 16S rRNA gene was sequenced to detect ASVs, enabling analysis of taxonomic composition at both genus and species levels. Analysis of 129 CHB samples yielded 684 ASVs, comprising 505 known species, 78 potentially new species, and 101 potentially higher taxa (Figure 2A). Species classification was based on representative 16S rRNA sequences showing identical or near-identical matches (>98.7% identity) with known type strain sequences (Parte, 2014). ASVs representing independent lineages within known genera were designated as potentially new species (Yarza et al., 2014). The 101 potentially higher taxa showed close affiliation with known genera, families, orders, or higher-level groups, suggesting new taxonomic ranks. The HC cohort analysis revealed 635 ASVs, including 454 known species, 81 potentially new species, and 100 potentially higher taxa (Figure 2B). Notably, the majority of the microbiota was identified at the species level, with relative abundances of dominant taxa reaching 79.32% and 82.53% in CHB and HC, respectively.

### Low, Medium, and high prevalent bacteria groups

Based on bacterial prevalence (McNulty et al., 2011; Yang et al., 2020), ASVs from both cohorts were categorized into three groups: low prevalence (<10%), medium prevalence (10%–60%), and high prevalence (>60%). The analysis revealed: (i) the low-prevalence bacterial group (386 ASVs, representing 2.19% of total reads in CHB vs. 236 ASVs, 0.11% in HC); (ii) the medium-prevalence bacterial group (259 ASVs, 36.66% in CHB vs. 218 ASVs, 1.84% in HC); and (iii) the high-prevalence bacterial group (38 ASVs, 61.15% in CHB vs. 181 ASVs, 98.05% in HC) (Figure 2C).

### Probiotic, commensal, and potential pathogenic bacteria

To characterize the microbial species composition in CHB and HC groups, species were categorized as probiotic, commensal, or potentially pathogenic bacteria based on a literature review. Potential pathogenic bacteria were identified using a comprehensive list of human bacterial pathogens (Bartlett et al., 2022), which are associated with clinical infections or outbreaks. After excluding probiotics (bacteria beneficial to human health), the remaining species were classified as harmless commensal bacteria. Analysis revealed that CHB patients exhibited significantly higher numbers and relative abundance of pathogens (225 species, 22.80% vs. 185 species, 11.49% in HC) and lower commensal bacteria (242 species, 43.99% vs. 237 species, 64.35% in HC) (Figure 2D). Several enteropathogenic bacteria (i.e., *Bacteroides fragilis* and *Haemophilus parainfluenzae*) were enriched in the microbiota of CHB patients (Supplementary Tables 1, 2). Additionally, certain potential pathogens (i.e., *Veillonella atypica* and *Veillonella parvula*) demonstrated notably high abundance in some CHB samples (Figure 2D). No significant differences in probiotics were observed between groups. These findings suggest that CHB patients possess a fecal microbiota enriched in pathogenic bacteria, potentially increasing susceptibility to opportunistic infections.

### Microbial diversity and composition in CHB individuals differ significantly from healthy individuals and exhibited the presence of two distinct enterotypes

**FIGURE 3:**
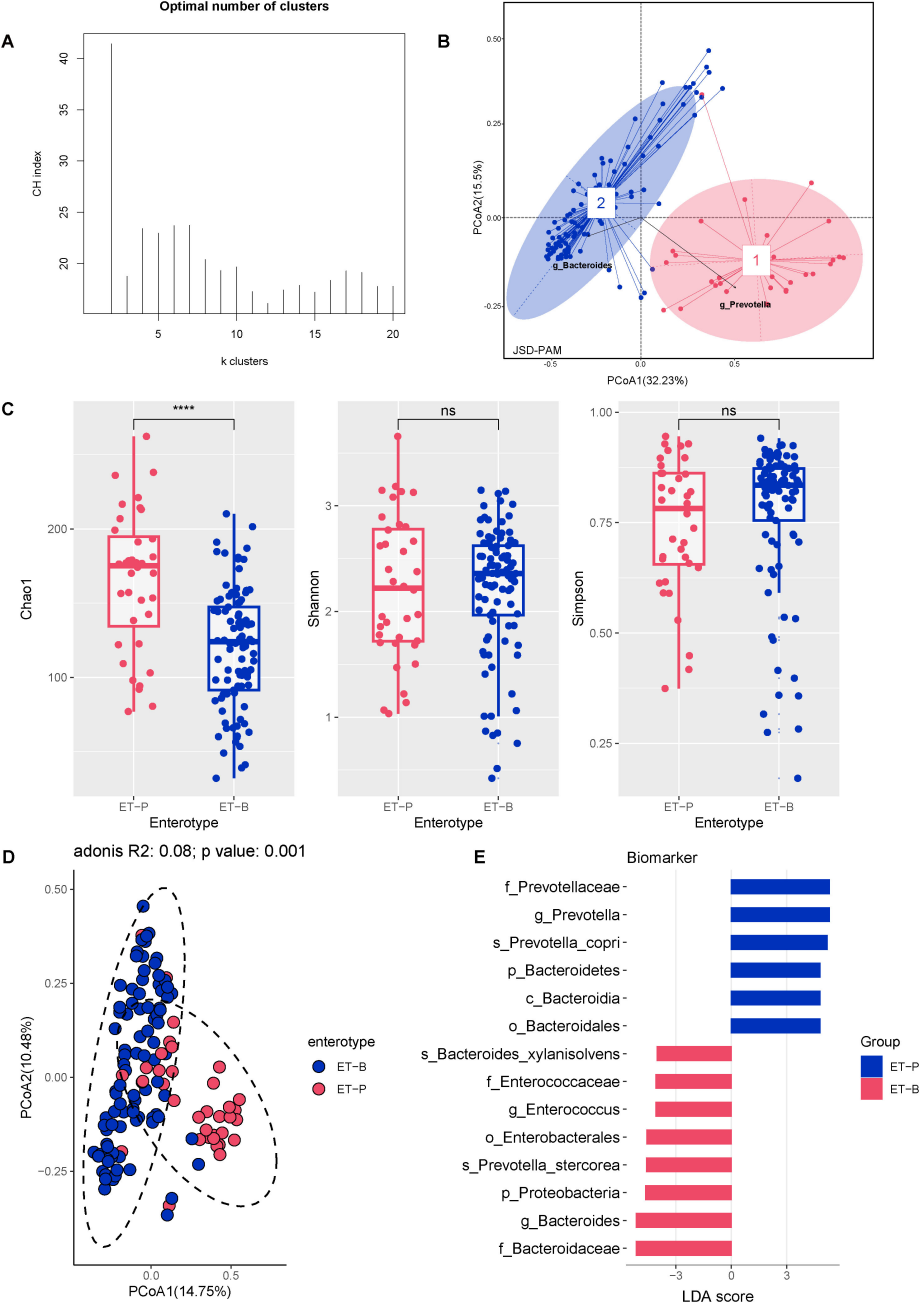
The CHB cohort exhibited two distinct enterotypes. (A) Calinski–Harabasz (CH) index of the PAM clustering algorithm. (B) PCoA plot based on Jensen–Shannon divergence (JSD) distance and PAM clustering for enterotype classification. **(C)** Alpha diversity of the two enterotypes, including Chao1 richness, Shannon index, and Simpson index. Significance codes: ****P < 0.0001. **(D)** Beta diversity of the two enterotypes. **(E)** Differential gut bacterial taxa between the two enterotypes determined by LEfSe, with results plotted at a threshold of log LDA score > 3.5 and *P* < 0.05.

Comparative analysis showed substantial differences in gut microbiota diversity between CHB and HC individuals. CHB individuals had lower species counts (Chao1), reduced richness (Shannon index), and decreased diversity (Simpson index) compared with HC individuals, with notable differences in microbial composition (Supplementary Figures 1A, B). Based on compositional variations in CHB, dimensionality reduction was applied to the complex, multidimensional structure of the gut microbiota, and enterotypes were constructed at the genus level by calculating the Jensen-Shannon distance and applying the PAM clustering algorithm. The CH index indicated that the optimal number of clusters for the CHB cohort (*N* = 129) was two, yielding distinct microbial clusters that were clearly separated in a PCoA plot (Figures 3A, B). Cluster 1 (*N* = 36) was characterized by *Prevotella* (ET-P), while cluster 2 (*N* = 93) was dominated by *Bacteroides* (ET-B). The diversity comparison revealed that ET-P exhibited higher richness compared with ET-B (Chao1 richness: *P* < 0.001) (Figures 3C, D). Biomarkers based on LEfSe for ET-P included *Prevotella* (LDA score 5.31, *P* < 0.001), *Prevotella copri* (LDA score 5.18, *P* < 0.001), *Faecalibacterium*, *Faecalibacterium prausnitzii*, and *Ruminococcus albus*. In contrast, ET-B was characterized by *Bacteroides* (LDA score 5.15, *P* < 0.001), *Prevotella stercorea* (LDA score 4.58, *P* < 0.01), *Enterococcus*, *Bacteroides xylanisolvens*, and *Fusobacterium* (Figure 3E).

Kyoto Encyclopedia of Genes and Genomes Orthology (KO) annotation was conducted to predict and compare gut microbiota functions between HC and CHB groups for the two enterotypes. ET-P showed significantly higher predicted functional potential in Genetic Information Processing than ET-B. Within the Metabolism main class, ET-P displayed significantly higher abundance in nucleotide metabolism, metabolism of cofactors and vitamins, and amino acid metabolism compared with ET-B, while carbohydrate metabolism was elevated in ET-B (Supplementary Figure 2). According to Enzyme Commission (EC) numbers, enzymes are classified into six categories: EC-1: oxidoreductases; EC-2: transferases; EC-3: hydrolases; EC-4: lyases; EC-5: isomerases; and EC-6: ligases. EC analysis of the two enterotypes revealed that ET-P exhibited significantly higher abundance levels for the top 25 enzymes across all six categories compared with ET-B (Supplementary Figure 2).

### Clinical indicators of CHB vary between two enterotypes, and enterosignatures predict disease progression and immune status

Comparison of clinical indicators between the two enterotypes in CHB patients revealed that ET-P exhibited significantly elevated levels of HBV DNA, HBsAg, and HBeAg. Furthermore, ET-P demonstrated significantly higher CD4^+^T-cell count, CD8^+^T-cell count, and alpha-fetoprotein (AFP) levels. Conversely, cancer antigen 125 (CA125) and carcinoembryonic antigen (CEA) levels were elevated in ET-B (Table 1).

**TABLE 1:** Comparison of epidemiological and clinical indicators between two enterotypes.

Characteristics	ET-P (*N* = 36)	ET-B (*N* = 93)	*P*-value
Age, years	43.5 (33.25–54.75)	53 (47–58)	0.003*
Male, *n* (%)	23 (63.90%)	67 (72.00%)	0.087
HBV DNA, IU/mL	6.28E05 (1.22E02–4.09E07)	9.90E01 (9.90E01–3.60E03)	0.006*
HBsAg, IU/mL	453.49 (251.00–3427.38)	251.00 (129.00–1174.89)	0.031*
HBsAb, mIU/mL	0.26 (0–0.79)	0.04 (0–0.42)	0.196
HBeAg, S/CO	4.01 (0.52–444.45)	0.54 (0.44–2.12)	0.015*
HBeAb, S/CO	1.20 (0.06–20.10)	0.56 (0.02–1.44)	0.031*
HBcAb, S/CO	9.28 (8.72–10.23)	9.51 (8.69–10.20)	0.705
HBcAbIgM, S/CO	0.23 (0.14–0.37)	0.12 (0.08–0.31)	0.011*
CD4, cell/uL	546.00 (432.25–814.25)	408.50 (234.25–632.25)	0.007*
CD8, cell/uL	312.00 (158.50–475.25)	212.00 (85.50–367.5)	0.014*
CD4/CD8 ratio	1.94 (1.47–2.46)	2.09 (1.39–2.92)	0.584
IgA, g/L	2.97 (1.90–3.52)	3.44 (2.57–4.05)	0.131
IgG, g/L	15.90 (13.78–20.35)	15.90 (11.50–19.90)	0.640
IgM, g/L	1.13 (0.83–1.76)	1.07 (0.64–1.62)	0.435
CRP, mg/L	2.00 (2.00–3.74)	2.00 (2.00–7.36)	0.337
CA-125, U/mL	12.01 (9.11–20.56)	23.94 (11.04–67.51)	0.023*
CA15-3, U/mL	9.25 (6.62–13.24)	10.87 (8.42–15.56)	0.051
CA19-9, U/mL	36.19 (15.08–61.11)	17.22 (12.67–49.10)	0.247
AFP, ng/mL	8.21 (2.52–154.70)	4.21 (1.87–14.22)	0.026*
CEA, ng/mL	2.00 (1.23–2.96)	2.42 (1.84–3.20)	0.035*
PCT, ng/mL	0.15 (0.02–0.27)	0.10 (0.03–0.28)	0.934

Numerical data were presented as median (interquartile range), comparison between two enterotypes was assessed by Mann-Whitney U test. Categorical variables were presented in *n* (%), and differences were assessed by Fisher’s exact tests. HBsAg, hepatitis B surface antigen; HBsAb, hepatitis B surface antibody; HBeAg, hepatitis B e antigen; HBeAb, hepatitis B e antibody; HBcAb, hepatitis B core antibody; HBcAbIgM, hepatitis B core antibody IgM; CD4, CD4^+^T-cell count; CD8, CD8^+^T-cell count; IgA, immunoglobulin A; IgG, immunoglobulin G; IgM, immunoglobulin M; CRP, C-reactive protein; CA-125, carbohydrate antigen 125; CA15-3, carbohydrate antigen 15-3; CA19-9, carbohydrate antigen 19-9; AFP, alpha-fetoprotein; CEA, carcinoembryonic antigen; PCT, procalcitonin.

Significance codes: *P < 0.05.

Differential signatures between enterotypes were identified using LEfSe with LDA score > 3, and a random forest classification model was constructed to determine significant signatures. The intersection of these methods yielded 15 enterosignatures: ET-P signatures included *Prevotella copri*, *Barnesiella intestinihominis*, Mollicutes, *Roseburia inulinivorans*, *Clostridiaceae*, and *Flavonifractor plautii*; ET-B signatures comprised *Catenibacterium mitsuokai*, *Christensenellaceae*, *Roseburia cecicola*, *Bacteroides xylanisolvens*, *Eubacterium ramulus*, Bacteroidales, *Ruminococcus gnavus*, and *Prevotella stercorea*.

**FIGURE 4:**
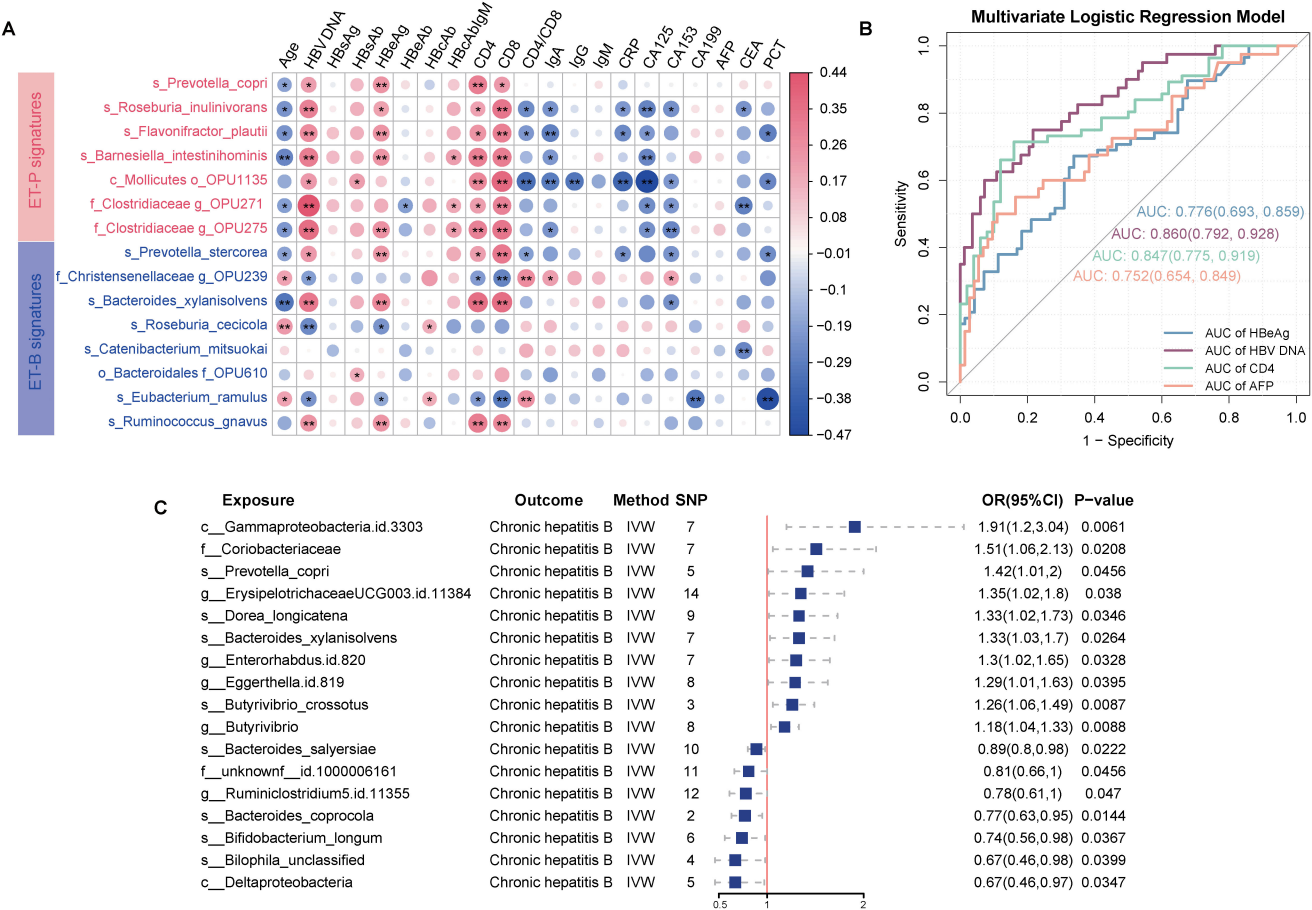
Predictive capability of enterosignatures for clinical indicators and causal relationships between gut microbiota and CHB. **(A)** Correlation heatmap between enterosignatures and clinical indicators. Valid associations were filtered at *P* < 0.05 after Benjamini–Hochberg correction. Significance codes: *P < 0.05; **P < 0.01. **(B)** ROC curves of multivariate logistic regression models predicting clinical phenotypes (HBeAg, HBV DNA, CD4^+^T-cell count, AFP) in CHB patients. HBeAg was grouped into HBeAg-positive and HBeAg-negative. HBV DNA levels were stratified at a threshold of 10**^4^** IU/mL. CD4^+^T-cell count were grouped into high and low immune states using a cutoff of 500 cells/μL. AFP was categorized into positive (>9 ng/mL) and negative (≤9 ng/mL) groups. **(C)** Forest plot illustrating significant causal associations between gut microbiota and CHB using the inverse variance weighted method, with *P* < 0.05.

Spearman correlation analysis revealed significant associations between enterosignatures and clinical indicators. Notably, ET-P-associated bacteria showed positive correlations with HBV DNA, HBeAg, CD4^+^T-cell count, and CD8^+^T-cell count, with correlation patterns varying significantly across enterotypes. These findings suggest that distinct microbial signatures between enterotypes may function as predictors of clinical indicators and CHB progression (Figure 4A).

To evaluate the predictive capacity of enterotype signatures for clinical indicators, HBeAg status was categorized into HBeAg-positive (HBeAg-P) and HBeAg-negative (HBeAg-N) groups. HBV DNA levels were stratified using a threshold of 10^4^ IU/mL (Tong and Trieu, 2013), CD4^+^T-cell count were classified into high and low immune states using a 500 cells/μL cutoff (Pantazis et al., 2023), and AFP was categorized into positive (>9 ng/mL) and negative (≤9 ng/mL) based on standard clinical reference values. Multivariate logistic regression models incorporating enterosignatures were developed, with age and sex as covariates to control for potential confounding effects. The models demonstrated predictive accuracy with area under the curve (AUC) values of 0.78 (95% CI: 0.69–0.86) for HBeAg status, 0.86 (95% CI: 0.79–0.93) for HBV DNA levels, 0.75 (95% CI: 0.65–0.84) for AFP status, and 0.85 (95% CI: 0.77–0.92) for CD4^+^T-cell count status. These results indicate that enterosignatures may serve as effective predictive biomarkers for various CHB-related clinical indicators (Figure 4B).

### Genome-wide mendelian randomization analysis identifies 16 species-level gut microbial taxa and potential mediators linking the gut microbiota to CHB outcomes

While significant correlations were established between gut microbiota composition and clinical indicators of CHB, these observational associations cannot establish causality. To address this limitation, MR analysis was employed to infer potential causal effects of gut microbial features on CHB risk and to explore mediating mechanisms involving metabolites. The integration of two gut microbiota datasets provided a comprehensive collection of 330 gut microbiota taxonomic groups, comprising 9 phyla, 16 classes, 20 orders, 43 families (including 3 unknown), 144 genera (including 12 unknown), and 98 species. Furthermore, 3,804 SNPs meeting a stringent genome-wide significance threshold of *P* < 1 × 10^–5^ were identified for these 330 bacterial taxa and considered as potential instrumental variables (IVs).

As shown in Supplementary Tables 3–9, the results indicated that 16 bacterial taxa demonstrated a causal relationship with CHB. Ten species exhibited positive causal relationships with CHB. Among these, several taxa were significantly associated with CHB: positively associated taxa included *Prevotella copri* (OR = 1.42, 95% CI = 1.01–2.00, *P* = 0.045), *Erysipelotrichaceae* UCG-003 ID 11384 (OR = 1.35, 95% CI = 1.02–1.80, *P* = 0.038), *Dorea longicatena* (OR = 1.33, 95% CI = 1.02–1.73, *P* = 0.035), *Bacteroides xylanisolvens* (OR = 1.33, 95% CI = 1.03–1.70, *P* = 0.033), *Eggerthella* (OR = 1.30, 95% CI = 1.01–1.67, *P* = 0.039), *Enterorhabdus* ID 820 (OR = 1.30, 95% CI = 1.02–1.65, *P* = 0.033), *Butyrivibrio crossotus* (OR = 1.26, 95% CI = 1.06–1.49, *P* = 0.009), and *Butyrivibrio* (OR = 1.18, 95% CI = 1.04–1.33, *P* = 0.009). Conversely, negatively associated taxa included *Bacteroides salyersiae* (OR = 0.89, 95% CI = 0.80–0.98, *P* = 0.022), *Ruminiclostridium* 5 ID 11355 (OR = 0.78, 95% CI = 0.61–0.99, *P* = 0.047), *Bacteroides coprocola* (OR = 0.77, 95% CI = 0.63–0.95, *P* = 0.014), *Bifidobacterium longum* (OR = 0.74, 95% CI = 0.56–0.98, *P* = 0.037), and *Bilophila* unclassified (OR = 0.67, 95% CI = 0.46–0.98, *P* = 0.040). Thus, the MR analysis suggested a potential genetically determined causal relationship between gut microbiota and CHB (Figure 4C, Supplementary Figure 3).

### Potential mediators and underlying mechanisms between the gut microbiota, metabolites and CHB outcomes

**FIGURE 5:**
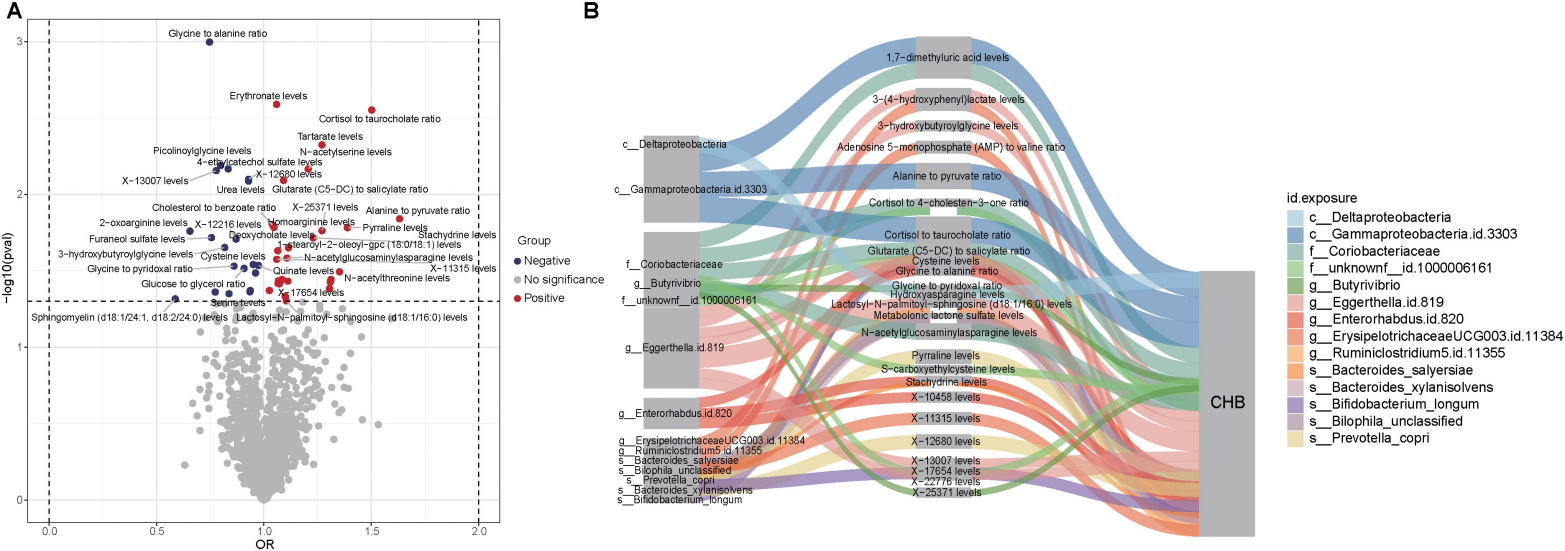
Causal relationships and potential mechanisms linking gut microbiota, metabolites, and CHB. (A) Volcano plot showing causal relationships between metabolites and CHB. Red dots represent metabolites with positive associations, while blue dots indicate metabolites with negative associations. (B) Sankey diagram illustrating potential mechanisms of interaction among gut microbiota, metabolites, and CHB.

The study began with two-sample Mendelian randomization (TSMR) analyses to systematically evaluate the causal effects of 1,400 metabolites on CHB. This investigation identified 47 metabolites with significant causal associations with CHB, including nine unknown metabolites (Figure 5A). Subsequently, two-step MR analyses were conducted to investigate the causal relationships between gut microbiota and these significant metabolites (Supplementary Tables 10–13). For microbial taxa and metabolites that demonstrated consistent significance in the TSMR, MVMR was applied to estimate direct effects after adjusting for potential mediation. The mediation effects were then quantified based on the difference between the total and direct effects (Figure 5B).

The MVMR analysis revealed that eight gut microbial taxa exhibited indirect effects on CHB through metabolite-mediated pathways. After adjusting for these mediating metabolites, the direct causal effects of these microbes were no longer significant. Key findings include: *Eggerthella*.id.819 showed CHB risk effects potentially mediated through five metabolites–3-hydroxybutyroylglycine (mediation proportion: 15.22%), glycine-to-alanine ratio (22.47%), lactosyl-N-palmitoyl-sphingosine (d18:1/16:0) (59.51%), X-13007 (84.34%), and X-22776 (1.85%). *Enterorhabdus*.id.820 showed harmful effects mediated by glycine-to-alanine ratio (69.24%), stachydrine (53.48%), and X-10458 (68.09%). *Bilophila* unclassified exhibited a protective effect against CHB that was completely attenuated after accounting for metabolonic lactone sulfate (mediation proportion: 14.02%) (Table 2).

**TABLE 2:** Multivariable Mendelian randomization (MVMR) reveals the potential mechanisms among gut microbiota, metabolites and CHB.

Exposure	Mediator	Outcome	Total effect	SNP number	Direct effect	Se	95% CI	*P*-value	Indirect effect	Mediated_proportion
c__Deltaproteobacteria	Hydroxyasparagine levels	CHB	−0.405	13	−0.279	0.187	−0.646, 0.088	0.136	−0.126	31.043
c__Gammaproteobacteria.id.3303	1,7-dimethyluric acid levels	CHB	0.649	10	0.124	0.318	−0.498, 0.747	0.696	0.525	80.894
c__Gammaproteobacteria.id.3303	Alanine to pyruvate ratio	CHB	0.649	11	0.089	0.237	−0.375, 0.554	0.706	0.560	86.287
c__Gammaproteobacteria.id.3303	Cortisol to taurocholate ratio	CHB	0.649	12	0.079	0.271	−0.453, 0.611	0.77	0.570	87.827
f__unknownf__id.1000006161	Cortisol to 4-cholesten-3-one ratio	CHB	−0.208	17	−0.035	0.104	−0.238, 0.168	0.736	−0.173	83.147
f__unknownf__id.1000006161	Glycine to alanine ratio	CHB	−0.208	17	−0.028	0.114	−0.252, 0.195	0.804	−0.180	86.518
f__unknownf__id.1000006161	S-carboxyethylcysteine levels	CHB	−0.208	17	0.033	0.106	−0.175, 0.241	0.753	−0.241	115.890
f__unknownf__id.1000006161	X-22776 levels	CHB	−0.208	14	−0.03	0.104	−0.233, 0.173	0.774	−0.178	85.555
g__*Eggerthella*.id.819	3-hydroxybutyroylglycine levels	CHB	0.262	8	0.222	0.119	−0.012, 0.456	0.063	0.040	15.216
g__*Eggerthella*.id.819	Glycine to alanine ratio	CHB	0.262	10	0.203	0.124	−0.041, 0.447	0.104	0.059	22.472
g__*Eggerthella*.id.819	Lactosyl-N-palmitoyl-sphingosine (d18:1/16:0) levels	CHB	0.262	16	0.106	0.127	−0.143, 0.355	0.405	0.156	59.517
g__*Eggerthella*.id.819	X-13007 levels	CHB	0.262	16	0.041	0.12	−0.195, 0.277	0.736	0.221	84.342
g__*Eggerthella*.id.819	X-22776 levels	CHB	0.262	7	0.257	0.131	−0.001, 0.514	0.051	0.005	1.849
g__Enterorhabdus.id.820	Glycine to alanine ratio	CHB	0.260	14	0.08	0.121	−0.157, 0.318	0.507	0.180	69.241
g__Enterorhabdus.id.820	Stachydrine levels	CHB	0.260	10	0.121	0.125	−0.124, 0.366	0.332	0.139	53.477
g__Enterorhabdus.id.820	X-10458 levels	CHB	0.260	14	0.083	0.113	−0.139, 0.305	0.464	0.177	68.087
g__ErysipelotrichaceaeUCG003.id.11384	3-(4-hydroxyphenyl) lactate levels	CHB	0.303	23	0.162	0.146	−0.124, 0.448	0.266	0.141	46.514
g__ErysipelotrichaceaeUCG003.id.11384	Adenosine 5′-monophosphate (AMP) to valine ratio	CHB	0.303	21	0.153	0.152	−0.144, 0.450	0.313	0.150	49.485
g__ErysipelotrichaceaeUCG003.id.11384	X-11315 levels	CHB	0.303	19	0.157	0.142	−0.121, 0.435	0.269	0.146	48.165
g__*Ruminiclostridium*5.id.11355	Glutarate (C5-DC) to salicylate ratio	CHB	−0.250	14	−0.106	0.126	−0.353, 0.140	0.399	−0.144	57.648
g__*Ruminiclostridium*5.id.11355	Lactosyl-N-palmitoyl-sphingosine (d18:1/16:0) levels	CHB	−0.250	20	−0.08	0.13	−0.335, 0.174	0.537	−0.170	68.036
s__*Bilophila*_unclassified	Metabolonic lactone sulfate levels	CHB	−0.399	12	−0.343	0.215	−0.763, 0.078	0.11	−0.056	14.018
f__Coriobacteriaceae	1,7-dimethyluric acid levels	CHB	0.410	8	0.524	0.238	0.058, 0.991	0.028	−0.114	−27.783
f__Coriobacteriaceae	Cortisol to 4-cholesten-3-one ratio	CHB	0.410	10	0.557	0.223	0.120, 0.994	0.012	−0.147	−35.830
f__Coriobacteriaceae	Cortisol to taurocholate ratio	CHB	0.410	10	0.506	0.206	0.102, 0.910	0.014	−0.096	−23.393
f__Coriobacteriaceae	N-acetylglucosaminylasparagine levels	CHB	0.410	12	0.505	0.207	0.099, 0.910	0.015	−0.095	−23.149
g__*Butyrivibrio*	Glycine to pyridoxal ratio	CHB	0.164	11	0.174	0.062	0.052, 0.296	0.005	−0.010	−6.191
g__*Butyrivibrio*	X-25371 levels	CHB	0.164	18	0.147	0.062	0.025, 0.268	0.018	0.017	10.287
g__*Eggerthella*	3-(4-hydroxyphenyl) lactate levels	CHB	0.262	12	0.258	0.126	0.012, 0.504	0.04	0.004	1.467
g__*Eggerthella*	Cortisol to taurocholate ratio	CHB	0.262	9	0.278	0.127	0.030, 0.527	0.028	−0.016	−6.171
s__*Bacteroides_salyersiae*	Stachydrine levels	CHB	−0.119	9	−0.153	0.069	−0.288, −0.018	0.026	0.034	−29.090
s__*Bacteroides_xylanisolvens*	Metabolonic lactone sulfate levels	CHB	0.282	16	0.293	0.117	0.063, 0.523	0.013	−0.011	−4.020
s__*Bifidobacterium_longum*	Cysteine levels	CHB	−0.298	8	−0.302	0.148	−0.593, −0.011	0.042	0.004	−1.375
s__*Bifidobacterium_longum*	X-17654 levels	CHB	−0.298	14	−0.298	0.139	−0.570, −0.026	0.032	0.000	−0.033
s__*Bilophila*_unclassified	Cortisol to taurocholate ratio	CHB	−0.399	9	−0.599	0.207	−1.005, −0.194	0.004	0.200	−50.155
s__*Prevotella_copri*	Pyrraline levels	CHB	0.349	10	0.317	0.142	0.040, 0.594	0.025	0.032	9.198
s__*Prevotella_copri*	X-12680 levels	CHB	0.349	11	0.386	0.145	0.101, 0.671	0.008	−0.037	−10.567

## Discussion

This study characterized the gut microbiota at the species level in patients with CHB and healthy controls using a data-driven approach based on the Human Gut Microbiome Analysis Database (HGMAD) for species-level characterization. The research established two enterotypes (ET-P and ET-B) and elucidated the correlations between enterotypes and clinical indicators in CHB patients. The representative species of these two enterotypes effectively predicted viral load, immune cells, HBeAg, and the liver cancer marker AFP. Through TSMR and MVMR analyses, the study established genetic causal relationships and explored potential mechanisms linking gut microbiota, 1,400 metabolites, and CHB. From a genetic perspective, this approach validated the causal associations between enterosignatures and CHB. The findings highlight the predictive potential of enterosignatures for clinical outcomes while underscoring the genetic causal links between gut microbiota, metabolites, and CHB, suggesting new possibilities for targeted therapeutic interventions and personalized treatment strategies.

The classification of high-, medium-, and low-prevalence bacterial groups was used to describe heterogeneity (Pu et al., 2023). The results demonstrated that in CHB patients, the high-prevalence bacterial group comprised 61.15% of total reads, whereas in HC individuals, it constituted 98.05%. These findings suggested that HBV infection induced significant alterations in the gut microbiome, with marked individual variability, whereas the gut microbiome in HC individuals maintained relative stability. Moreover, potential pathogenic bacteria represented 22.80% of total reads in CHB patients, compared with 11.49% in HC individuals. These observations indicated that HBV infection resulted in substantial modifications to the gut microbiome, characterized by enhanced individual variability and an elevated proportion of pathogenic bacteria compared with healthy individuals (Zeng et al., 2020).

Significant differences in microbiota diversity and composition existed between CHB and HC groups, aligning with previously reported findings in the literature. The microbial diversity in CHB was notably lower than in HC, potentially attributable to multiple factors, including varying stages of HBV infection, diverse immune states, and different levels of viral replication (Long et al., 2023).

Given the heterogeneity in the microbial composition of CHB, we performed dimensionality reduction and clustering analysis of the complex gut microbiota to identify enterotypes and examine their association with CHB. Enterotypes have been shown to remain stable across sex, cultural background, and geographical factors (Costea et al., 2017). The analysis revealed two distinct enterotypes in CHB: ET-P and ET-B, dominated by *Prevotella* and *Bacteroides*, respectively. This classification aligned with enterotype characteristics observed in the Chinese population and corroborated previous findings (Liang et al., 2017). ET-P demonstrated significantly higher species richness compared with ET-B.

With respect to metabolic pathways, ET-P exhibited elevated functional potential in nucleotide metabolism, cofactor and vitamin metabolism, and amino acid metabolism relative to ET-B. Conversely, carbohydrate metabolism was enhanced in ET-B, consistent with known *Bacteroides* functions (Cantarel et al., 2009). Among the six major enzyme classes–oxidoreductases, transferases, hydrolases, lyases, isomerases, and ligases–ET-P displayed significantly higher levels than ET-B, indicating more active enzyme metabolism that reflected metabolic differences between enterotype signatures (Wright et al., 2000).

Clinical serological indicators demonstrated higher HBV DNA levels and HBeAg replication activity in ET-P compared with ET-B, supporting the hypothesis that *Prevotella* influences gut antiviral defense mechanisms (Glavan et al., 2016). ET-P also showed significantly elevated CD4^+^T-cell count and CD8^+^T-cell count, consistent with studies in HIV patients and those with periodontal disease (Pinacchio et al., 2020), where *Prevotella* abundance correlated positively with CD4^+^T-cell count and CD8^+^T-cell count. This suggested that *Prevotella* drove sustained T-cell activation and inflammation, primarily through TLR2 signaling, which stimulated antigen-presenting cells to produce Th17-polarizing cytokines, including IL-23 and IL-1. Additionally, *Prevotella* stimulated epithelial cells to secrete IL-8, IL-6, and CCL20, enhancing mucosal Th17 immune responses and neutrophil recruitment. These *Prevotella*-induced responses intensified mucosal inflammation, potentially leading to systemic dissemination of inflammatory mediators, bacterial components, and intact bacteria, which may have contributed to broader inflammatory disease outcomes (Vázquez-Castellanos et al., 2015; Larsen, 2017).

Alpha-fetoprotein levels were significantly higher in ET-P compared with ET-B, consistent with findings from liver cancer patients showing positive associations between AFP levels and *Prevotella* abundance (Zhang W. et al., 2023). Further analysis of enterotype–clinical indicator relationships revealed that the ET-P enterosignature correlated positively with CHB-specific antigens and CD4^+^/CD8^+^ T-cell counts. Beyond *Prevotella copri*, ET-P representative species such as *Barnesiella intestinihominis* co-occurred with Enterobacteriaceae, suggesting potential synergistic effects in establishing pro-inflammatory gut conditions (Tsakmaklis et al., 2023). *Flavonifractor plautii* was associated with increased IL-10 expression and CD4^+^CD25^+^ cell expansion in splenocytes, suggesting immunoregulatory functions (Ogita et al., 2020).

Bacteroides demonstrated distinct functional characteristics. *Prevotella stercorea* was suggested to enhance androgen deprivation therapy efficacy by opposing microbiota that promote castration-resistant prostate cancer, potentially through mechanisms stabilizing microbial ecology and immune homeostasis (Pernigoni et al., 2021). Additionally, *Eubacterium ramulus* enrichment in afebrile COVID-19 patients inversely correlated with inflammatory markers, indicating potential protective effects. These observations supported enterotype-specific immunovirological signatures in CHB (Zhou et al., 2021). Such enterosignatures may serve as biomarkers for predicting viral replication activity, immune status, and clinical progression.

Studies by Zheng et al. (2023) demonstrated the potential of gut microbes as biomarkers for early recurrence prediction and treatment response in HBV-related hepatocellular carcinoma, and as prognostic indicators for HBV-related acute-on-chronic liver failure (Wang et al., 2021). Multivariate logistic regression analysis and ROC curve evaluation revealed that enterosignatures effectively distinguished HBV DNA loads, viral replication status, immune status, and outcome indicators. Traditional approaches had been insufficient for comprehensively analyzing microbiota–disease relationships due to the complexity of the gut microbiota. The enterotype concept, involving dimensionality reduction and clustering of gut compositional patterns (Arumugam et al., 2011), revealed numerous associations between enterotypes and disease phenotypes. Increased *Prevotella* or ET-P abundance correlated with rheumatoid arthritis, type II diabetes, and HIV, while elevated *Bacteroides* levels were associated with non-alcoholic steatohepatitis (Zhu et al., 2013) and immune senescence (Le Chatelier et al., 2013).

Enterotypes may enhance microbiota-based diagnostics, therapies, and disease prevention, supporting personalized interventions. Caenepeel et al. (2024) demonstrated associations between enterotypes and biological therapy outcomes in inflammatory bowel disease, while Zhu et al. (2024) identified an aging-enriched enterotype linked to improved immunotherapy outcomes in older patients, confirmed through FMT experiments.

While associations between gut microbiota and clinical indicators have been established, correlation did not necessarily indicate causation, and establishing causal relationships required additional analysis. This study employed TSMR to investigate the genetic relationships between gut microbiota, metabolites, and CHB. Given that metabolites play a crucial role in the development of liver inflammation (Tang et al., 2022), the MVMR analysis confirmed their mediating roles, with specific microbiota classes demonstrating substantial mediation effects.

Through combined analysis of gut microbiota correlations and causal relationships, our findings revealed that certain gut microbial taxa could influence CHB progression through both facilitating and mitigating effects. Notably, elevated abundance of *Prevotella copri* correlated with increased CHB risk, suggesting its potential utility as a predictive biomarker and therapeutic target for CHB management. This finding is consistent with previous research indicating that CHB patients with ET-P generally exhibit higher viral loads, active viral replication, enhanced immune responses, and a potentially elevated risk of hepatocellular carcinoma (Zhang Q. et al., 2023). These results underscore the potential value of enterotype-based stratification for personalizing treatment strategies. Specifically, ET-P patients, characterized by a predominance of *Prevotella copri*, may constitute a distinct subgroup with higher HBV viral load, more active viral replication, and increased risk of hepatocellular carcinoma, necessitating specialized monitoring and aggressive therapeutic measures. However, it should be emphasized that gut microbial biomarkers should not be viewed as entirely independent of established clinical parameters, nor should they serve as a standalone gold standard. Instead, they should be integrated with conventional clinical indicators to enable a comprehensive evaluation of disease status. That said, the advantages of gut microbiota analysis for non-invasive testing are substantial. It demonstrates considerable promise as an early-warning tool during initial disease onset and progression, thereby offering innovative perspectives and strategies for disease prevention and control.

Additionally, MR analysis identified *Bacteroides xylanisolvens* in ET-B as causally associated with CHB. Through MVMR-based mediation analysis, we identified eight gut microbial taxa whose effects on CHB were mediated by metabolites. However, despite demonstrating causal associations with CHB, the representative species of ET-P (*Prevotella copri*) and ET-B (*Bacteroides xylanisolvens*) lacked clearly defined metabolite-mediated mechanistic pathways underlying their effects. In two recent studies, *Prevotella copri* was shown to significantly enrich the level of 5-aminopentanoic acid (5-AVA). 5-AVA aggravated palmitic acid-induced lipid accumulation in HepG2 cells and primary mouse hepatocytes, and it could promote the development of pediatric metabolic dysfunction-associated steatotic liver disease (MASLD) (Xu et al., 2025). Furthermore, these perturbations contributed to the exacerbation of hepatic fibrosis by disrupting lipid metabolism and impairing gut barrier integrity, which in turn promoted sustained inflammation and accelerated scarring within the liver tissue (Leitman et al., 2024).

Several limitations warranted consideration in our analysis. The cross-sectional study design limited our ability to collect comprehensive longitudinal data, constraining our understanding of how microbiota influenced CHB progression over time. Additionally, single-institution sampling and lack of mechanistic validation with resolved HBV infections restricted our capacity to draw more definitive conclusions regarding the long-term impacts of HBV resolution on gut microbiota and clinical outcomes. Despite these constraints, our findings provided valuable insights and established a foundation for future longitudinal studies with larger, more diverse cohorts.

In conclusion, this study demonstrated that species-level enterosignatures functioned as reliable predictors of infection status, immune response characteristics, and potential CHB-associated risks. Furthermore, we established a causal relationship between species-level gut microbiota composition, metabolites, and CHB, elucidating how specific microbial profiles influenced disease progression and outcomes. These findings enhanced our understanding of the gut–liver axis in HBV pathogenesis and provided a foundation for developing microbe-targeted therapies aimed at achieving complete hepatitis B virus clearance in CHB patients.

## Data Availability

The raw sequencing data generated in this study has been deposited in the National Microbiology Data Center (NMDC) with accession numbers NMDC10019892 (https://nmdc.cn/resource/genomics/project/detail/NMDC10019892). The data of MR study were derived from the following resources available in the public domain: GWAS Catalog, https://www.ebi.ac.uk/gwas/, IEU OpenGWAS project, https://gwas.mrcieu.ac.uk/, MiBioGen consortium, https://mibiogen.gcc.rug.nl.

## Footnotes

1
https://www.ebi.ac.uk/gwas/

## Supplementary material

Supplementary Figure 1 Diversity analysis of gut microbiota between CHB and HC. (A) Alpha diversity analysis. CHB patients exhibited reduced gut microbial species richness and diversity compared to HC, as evidenced by significantly lower values of the Chao1 index, Simpson index, and Shannon index. (B) Beta diversity analysis. Principal coordinates analysis (PCoA) based on Bray-Curtis distances revealed significant compositional dissimilarities in gut microbiota between CHB and HC groups (PERMANOVA: R^2^ = 0.06, *P* = 0.001). **P* < 0.05; ***P* < 0.01; ****P* < 0.001.

Supplementary Figure 2 Comparative functional annotation of enterotypes based on KEGG Orthology (KO) and Enzyme Commission (EC) profiles. (A) Differentially enriched KEGG pathways between the two enterotypes. Pathways showing significant disparities (threshold: *P* < 0.05) are ranked by effect size. (B) Top 25 EC categories with the most pronounced differences between enterotypes.

Supplementary Figure 3 The causal link between the taxonomy of 330 gut microbiota and CHB.

## References

[B1] Arumugam M. Raes J. Pelletier E. Le Paslier D. Yamada T. Mende D. R. (2011). Enterotypes of the human gut microbiome. *Nature* 473 174–180. 10.1038/nature09944 21508958 PMC3728647

[B2] Bartlett A. Padfield D. Lear L. Bendall R. Vos M. (2022). A comprehensive list of bacterial pathogens infecting humans. *Microbiology* 168:1269. 10.1099/mic.0.001269 36748702

[B3] Bokulich N. A. Subramanian S. Faith J. J. Gevers D. Gordon J. I. Knight R. (2013). Quality-filtering vastly improves diversity estimates from Illumina amplicon sequencing. *Nat. Methods* 10 57–59. 10.1038/nmeth.2276 23202435 PMC3531572

[B4] Bowden J. Davey Smith G. Burgess S. (2015). Mendelian randomization with invalid instruments: Effect estimation and bias detection through Egger regression. *Int. J. Epidemiol.* 44 512–525. 10.1093/ije/dyv080 26050253 PMC4469799

[B5] Bushnell B. Rood J. Singer E. (2017). BBMerge – Accurate paired shotgun read merging via overlap. *PLoS One* 12:e0185056. 10.1371/journal.pone.0185056 29073143 PMC5657622

[B6] Caenepeel C. Falony G. Machiels K. Verstockt B. Goncalves P. J. Ferrante M. (2024). Dysbiosis and associated stool features improve prediction of response to biological therapy in inflammatory bowel disease. *Gastroenterology* 166 483–495. 10.1053/j.gastro.2023.11.304 38096956

[B7] Cantarel B. L. Coutinho P. M. Rancurel C. Bernard T. Lombard V. Henrissat B. (2009). The Carbohydrate-Active EnZymes database (CAZy): An expert resource for glycogenomics. *Nucleic Acids Res.* 37 233–238. 10.1093/nar/gkn663 18838391 PMC2686590

[B8] Chen Y. Lu T. Pettersson-Kymmer U. Stewart I. D. Butler-Laporte G. Nakanishi T. (2023). Genomic atlas of the plasma metabolome prioritizes metabolites implicated in human diseases. *Nat. Genet.* 55 44–53. 10.1038/s41588-022-01270-1 36635386 PMC7614162

[B9] Chua H.-H. Chen Y.-H. Wu L.-L. Yang H.-C. Lin C.-R. Chen H.-L. (2023). Antagonism between gut *Ruminococcus gnavus* and *Akkermansia muciniphila* modulates the progression of chronic hepatitis B. *Cell Mol. Gastroenterol. Hepatol.* 17 361–381. 10.1016/j.jcmgh.2023.12.003 38092311 PMC10821531

[B10] Costea P. I. Hildebrand F. Arumugam M. Bäckhed F. Blaser M. J. Bushman F. D. (2017). Enterotypes in the landscape of gut microbial community composition. *Nat. Microbiol.* 3 8–16. 10.1038/s41564-017-0072-8 29255284 PMC5832044

[B11] Davey Smith G. Ebrahim S. (2003). ‘Mendelian randomization’: Can genetic epidemiology contribute to understanding environmental determinants of disease? *Int. J. Epidemiol.* 32 1–22. 10.1093/ije/dyg070 12689998

[B12] Deng Y. Yang K. Zhou G. Wang N. Liu C. Chen Z. (2023). Correlations of intestinal microorganisms with liver and immune functions of patients with human immunodeficiency virus and hepatitis B virus coinfection. *Afr. Health Sci.* 23 460–467. 10.4314/ahs.v23i3.53 38357144 PMC10862600

[B13] Douglas G. M. Maffei V. J. Zaneveld J. R. Yurgel S. N. Brown J. R. Taylor C. M. (2020). PICRUSt2 for prediction of metagenome functions. *Nat. Biotechnol.* 38 685–688. 10.1038/s41587-020-0548-6 32483366 PMC7365738

[B14] Frioux C. Ansorge R. Özkurt E. Ghassemi Nedjad C. Fritscher J. Quince C. (2023). Enterosignatures define common bacterial guilds in the human gut microbiome. *Cell Host Microbe* 31 1111–1125.e6. 10.1016/j.chom.2023.05.024 37339626

[B15] Gao Y. Zhang G. Jiang S. Liu Y. (2024). Wekemo bioincloud: A user-friendly platform for meta-omics data analyses. *Imeta* 3:e175. 10.1002/imt2.175 38868508 PMC10989175

[B16] Glavan T. W. Gaulke C. A. Santos Rocha C. Sankaran-Walters S. Hirao L. A. Raffatellu M. (2016). Gut immune dysfunction through impaired innate pattern recognition receptor expression and gut microbiota dysbiosis in chronic SIV infection. *Mucosal Immunol.* 9 677–688. 10.1038/mi.2015.92 26376368 PMC4794436

[B17] Jeng W.-J. Papatheodoridis G. V. Lok A. S. F. (2023). Hepatitis B. *Lancet* 401 1039–1052. 10.1016/S0140-6736(22)01468-4 36774930

[B18] Kurilshikov A. Medina-Gomez C. Bacigalupe R. Radjabzadeh D. Wang J. Demirkan A. (2021). Large-scale association analyses identify host factors influencing human gut microbiome composition. *Nat. Genet.* 53 156–165. 10.1038/s41588-020-00763-1 33462485 PMC8515199

[B19] Larsen J. M. (2017). The immune response to *Prevotella* bacteria in chronic inflammatory disease. *Immunology* 151 363–374. 10.1111/imm.12760 28542929 PMC5506432

[B20] Le Chatelier E. Nielsen T. Qin J. Prifti E. Hildebrand F. Falony G. (2013). Richness of human gut microbiome correlates with metabolic markers. *Nature* 500 541–546. 10.1038/nature12506 23985870

[B21] Leitman M. Zhang D. Pawar S. Shera S. Hernandez L. Jacobs J. P. (2024). The association between *Prevotella copri* and advanced fibrosis in the progression of metabolic dysfunction-associated steatotic liver disease. *BioRxiv [Preprint]* 10.1101/2024.11.22.624957 PMC12251637 40647253

[B22] Liang C. Tseng H.-C. Chen H.-M. Wang W.-C. Chiu C.-M. Chang J.-Y. (2017). Diversity and enterotype in gut bacterial community of adults in Taiwan. *BMC Genom.* 18:932. 10.1186/s12864-016-3261-6 28198673 PMC5310273

[B23] Liu Q. Li F. Zhuang Y. Xu J. Wang J. Mao X. (2019). Alteration in gut microbiota associated with hepatitis B and non-hepatitis virus related hepatocellular carcinoma. *Gut Pathog* 11:1. 10.1186/s13099-018-0281-6 30675188 PMC6337822

[B24] Long J. Gong J. Zhu H. Liu X. Li L. Chen B. (2023). Difference of gut microbiota between patients with negative and positive HBeAg in chronic hepatitis B and the effect of tenofovir alafenamide on intestinal flora. *Front. Microbiol.* 14:1232180. 10.3389/fmicb.2023.1232180 37799607 PMC10548823

[B25] Lopera-Maya E. A. Kurilshikov A. Van Der Graaf A. Hu S. Andreu-Sánchez S. Chen L. (2022). Effect of host genetics on the gut microbiome in 7,738 participants of the Dutch microbiome project. *Nat. Genet.* 54 143–151. 10.1038/s41588-021-00992-y 35115690

[B26] Lovmar L. Ahlford A. Jonsson M. Syvänen A.-C. (2005). Silhouette scores for assessment of SNP genotype clusters. *BMC Genom.* 6:35. 10.1186/1471-2164-6-35 15760469 PMC555759

[B27] McNulty N. P. Yatsunenko T. Hsiao A. Faith J. J. Muegge B. D. Goodman A. L. (2011). The impact of a consortium of fermented milk strains on the gut microbiome of gnotobiotic mice and monozygotic twins. *Sci. Transl. Med.* 3:106ra106. 10.1126/scitranslmed.3002701 22030749 PMC3303609

[B28] Noguera-Julian M. Rocafort M. Guillén Y. Rivera J. Casadellà M. Nowak P. (2016). Gut microbiota linked to sexual preference and HIV infection. *EBioMedicine* 5 135–146. 10.1016/j.ebiom.2016.01.032 27077120 PMC4816837

[B29] Ogita T. Yamamoto Y. Mikami A. Shigemori S. Sato T. Shimosato T. (2020). Oral administration of flavonifractor plautii strongly suppresses TH2 immune responses in mice. *Front. Immunol.* 11:379. 10.3389/fimmu.2020.00379 32184789 PMC7058663

[B30] Ott J. J. Stevens G. A. Groeger J. Wiersma S. T. (2012). Global epidemiology of hepatitis B virus infection: New estimates of age-specific HBsAg seroprevalence and endemicity. *Vaccine* 30 2212–2219. 10.1016/j.vaccine.2011.12.116 22273662

[B31] Pantazis N. Paparizos V. Papastamopoulos V. Metallidis S. Antoniadou A. Adamis G. (2023). Low pre-ART CD4 count is associated with increased risk of clinical progression or death even after reaching 500 CD4 cells/μL on ART. *PLoS One* 18:e0283648. 10.1371/journal.pone.0283648 36996018 PMC10062628

[B32] Parte A. C. (2014). LPSN—list of prokaryotic names with standing in nomenclature. *Nucl. Acids Res.* 42 D613–D616. 10.1093/nar/gkt1111 24243842 PMC3965054

[B33] Pernigoni N. Zagato E. Calcinotto A. Troiani M. Mestre R. P. Calì B. (2021). Commensal bacteria promote endocrine resistance in prostate cancer through androgen biosynthesis. *Science* 374 216–224. 10.1126/science.abf8403 34618582

[B34] Pinacchio C. Scagnolari C. Iebba V. Santinelli L. Innocenti G. P. Frasca F. (2020). High abundance of genus *Prevotella* is associated with dysregulation of IFN-I and T cell response in HIV-1-infected patients. *AIDS* 34 1467–1473. 10.1097/QAD.0000000000002574 32675560

[B35] Pu J. Yang J. Lu S. Jin D. Luo X. Xiong Y. (2023). Species-Level taxonomic characterization of uncultured core gut microbiota of plateau pika. *Microbiol. Spectr.* 11:e03495-22. 10.1128/spectrum.03495-22 37067438 PMC10269723

[B36] Sakaue S. Kanai M. Tanigawa Y. Karjalainen J. Kurki M. Koshiba S. (2021). A cross-population atlas of genetic associations for 220 human phenotypes. *Nat. Genet.* 53 1415–1424. 10.1038/s41588-021-00931-x 34594039 PMC12208603

[B37] Sohn K. Choi S. Jung J. Choi J. Cho S. Yi H. (2023). Different inflammatory features of asthma according to gut microbiome enterotype. *Allergy* 78 2997–3001. 10.1111/all.15768 37199247

[B38] Su Q. Lau R. I. Liu Q. Li M. K. T. Yan Mak J. W. Lu W. (2024). The gut microbiome associates with phenotypic manifestations of post-acute COVID-19 syndrome. *Cell Host Microbe* 32 651–660.e4. 10.1016/j.chom.2024.04.005 38657605

[B39] Tang J. Xiong K. Zhang T. Han H. (2022). Application of metabolomics in diagnosis and treatment of chronic liver diseases. *Crit. Rev. Anal. Chem.* 52 906–916. 10.1080/10408347.2020.1842172 33146026

[B40] Terrault N. A. Bzowej N. H. Chang K. Hwang J. P. Jonas M. M. Murad M. H. (2016). AASLD guidelines for treatment of chronic hepatitis B. *Hepatology* 63 261–283. 10.1002/hep.28156 26566064 PMC5987259

[B41] Tong M. J. Trieu J. (2013). Hepatitis B inactive carriers: Clinical course and outcomes. *J. Digest Dis.* 14 311–317. 10.1111/1751-2980.12051 23433008

[B42] Tsakmaklis A. Farowski F. Zenner R. Lesker T. R. Strowig T. Schlößer H. (2023). TIGIT+ NK cells in combination with specific gut microbiota features predict response to checkpoint inhibitor therapy in melanoma patients. *BMC Cancer* 23:1160. 10.1186/s12885-023-11551-5 38017389 PMC10685659

[B43] Vallet N. Salmona M. Malet-Villemagne J. Bredel M. Bondeelle L. Tournier S. (2023). Circulating T cell profiles associate with enterotype signatures underlying hematological malignancy relapses. *Cell Host Microbe* 31 1386–1403.e6. 10.1016/j.chom.2023.06.009 37463582

[B44] Vázquez-Castellanos J. F. Serrano-Villar S. Latorre A. Artacho A. Ferrús M. L. Madrid N. (2015). Altered metabolism of gut microbiota contributes to chronic immune activation in HIV-infected individuals. *Mucosal Immunol.* 8 760–772. 10.1038/mi.2014.107 25407519

[B45] Verbanck M. (2018). Detection of widespread horizontal pleiotropy in causal relationships inferred from Mendelian randomization between complex traits and diseases. *Nat. Genet.* 50 693–698. 10.1038/s41588-018-0099-7 29686387 PMC6083837

[B46] Wang K. Zhang Z. Mo Z.-S. Yang X.-H. Lin B.-L. Peng L. (2021). Gut microbiota as prognosis markers for patients with HBV-related acute-on-chronic liver failure. *Gut Microbes* 13:1921925. 10.1080/19490976.2021.1921925 34006193 PMC8143260

[B47] Wang M. Yuan T. Chen J. Yang J. Pu J. Lin W. (2025). A species-level identification pipeline for human gut microbiota based on the V3-V4 regions of 16S rRNA. *Front. Microbiol.* 16:1553124. 10.3389/fmicb.2025.1553124 40226098 PMC11985812

[B48] Wong D. K.-H. Seto W.-K. Fung J. Ip P. Huang F.-Y. Lai C.-L. (2013). Reduction of hepatitis B surface antigen and covalently closed circular DNA by Nucleos(t)ide analogues of different potency. *Clin. Gastroenterol. Hepatol.* 11 1004–1010.e1. 10.1016/j.cgh.2013.01.026 23376799

[B49] Wright D. P. Rosendale D. I. Roberton A. M. (2000). *Prevotella* enzymes involved in mucin oligosaccharide degradation and evidence for a small operon of genes expressed during growth on mucin. *FEMS Microbiol. Lett.* 190 73–79. 10.1111/j.1574-6968.2000.tb09265.x 10981693

[B50] Xu Q.-Y. Ren T.-Y. Zhou Y.-C. Xu J. Du L.-D. Hong D.-Y. (2025). Prevotella copri-produced 5-aminopentanoic acid promotes pediatric metabolic dysfunction-associated steatotic liver disease. *Hepatobil. Pancreatic Dis. Int.* 24 303–315. 10.1016/j.hbpd.2025.02.004 40057459

[B51] Yang J. Pu J. Lu S. Bai X. Wu Y. Jin D. (2020). Species-Level analysis of human gut microbiota with metataxonomics. *Front. Microbiol.* 11:2029. 10.3389/fmicb.2020.02029 32983030 PMC7479098

[B52] Yarza P. Yilmaz P. Pruesse E. Glöckner F. O. Ludwig W. Schleifer K.-H. (2014). Uniting the classification of cultured and uncultured bacteria and archaea using 16S rRNA gene sequences. *Nat. Rev. Microbiol.* 12 635–645. 10.1038/nrmicro3330 25118885

[B53] Zeng Y. Chen S. Fu Y. Wu W. Chen T. Chen J. (2020). Gut microbiota dysbiosis in patients with hepatitis B virus–induced chronic liver disease covering chronic hepatitis, liver cirrhosis and hepatocellular carcinoma. *J. Viral Hepatitis* 27 143–155. 10.1111/jvh.13216 31600845

[B54] Zhang Q. Zhou J. Zhang X. Mao R. Zhang C. (2023). Mendelian randomization supports causality between gut microbiota and chronic hepatitis B. *Front. Microbiol.* 14:1243811. 10.3389/fmicb.2023.1243811 37655340 PMC10467284

[B55] Zhang W. Xu X. Cai L. Cai X. (2023). Dysbiosis of the gut microbiome in elderly patients with hepatocellular carcinoma. *Sci. Rep.* 13:7797. 10.1038/s41598-023-34765-w 37179446 PMC10182990

[B56] Zheng C. Lu F. Chen B. Yang J. Yu H. Wang D. (2023). Gut microbiome as a biomarker for predicting early recurrence of HBV -related hepatocellular carcinoma. *Cancer Sci.* 114 4717–4731. 10.1111/cas.15983 37778742 PMC10728007

[B57] Zhou Y. Shi X. Fu W. Xiang F. He X. Yang B. (2021). Gut microbiota dysbiosis correlates with abnormal immune response in moderate COVID-19 patients with fever. *J. Inflammat. Res.* 14 2619–2631. 10.2147/JIR.S311518 34168484 PMC8217908

[B58] Zhu L. Baker S. S. Gill C. Liu W. Alkhouri R. Baker R. D. (2013). Characterization of gut microbiomes in nonalcoholic steatohepatitis (NASH) patients: A connection between endogenous alcohol and NASH. *Hepatology* 57 601–609. 10.1002/hep.26093 23055155

[B59] Zhu X. Huang X. Hu M. Sun R. Li J. Wang H. (2024). A specific enterotype derived from gut microbiome of older individuals enables favorable responses to immune checkpoint blockade therapy. *Cell Host Microbe* 32 489–505. 10.1016/j.chom.2024.03.002 38513657

