# Supplementary figures and images for: Species-level enterosignatures predict clinical phenotypes in chronic hepatitis B and causal triangulation of gut-metabolite-CHB interactions
Source: Front Microbiol. 2025 Oct 31;16:1683451. doi: 10.3389/fmicb.2025.1683451 (PMC12616741; doi:10.3389/fmicb.2025.1683451)

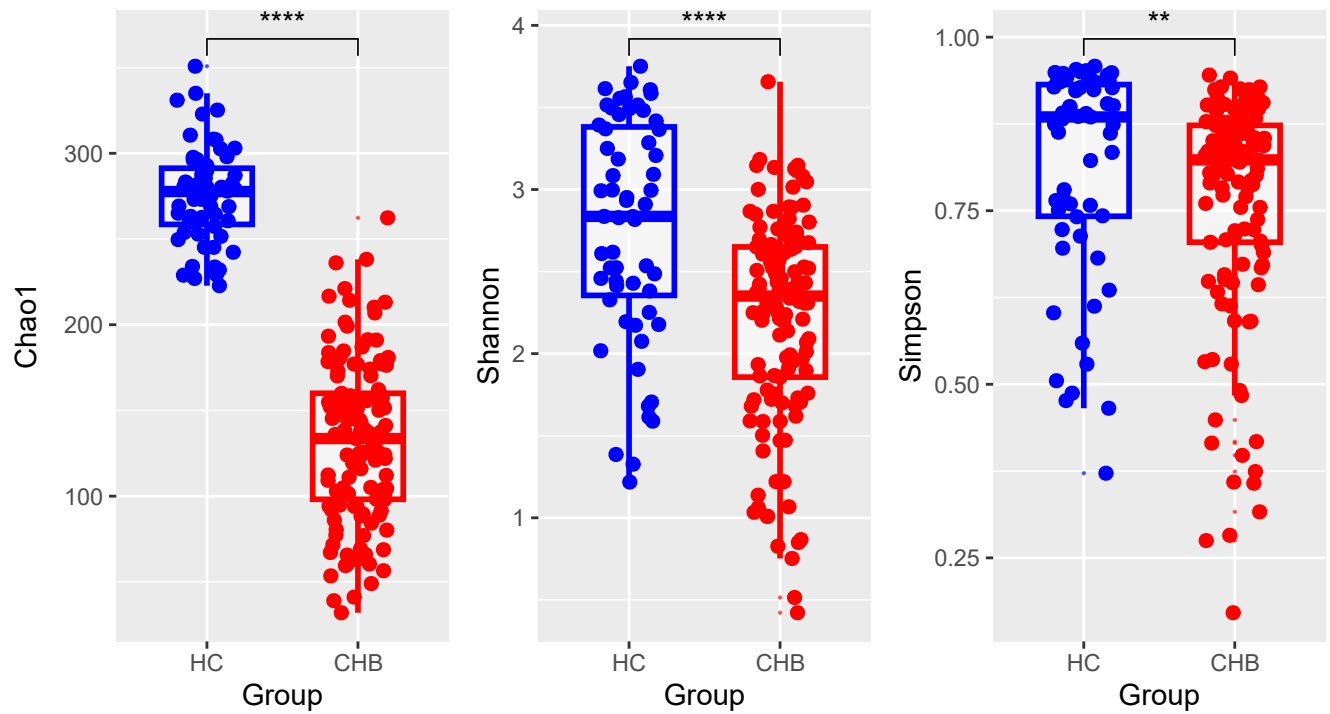

adonis R2: 0.06; p value: 0.001

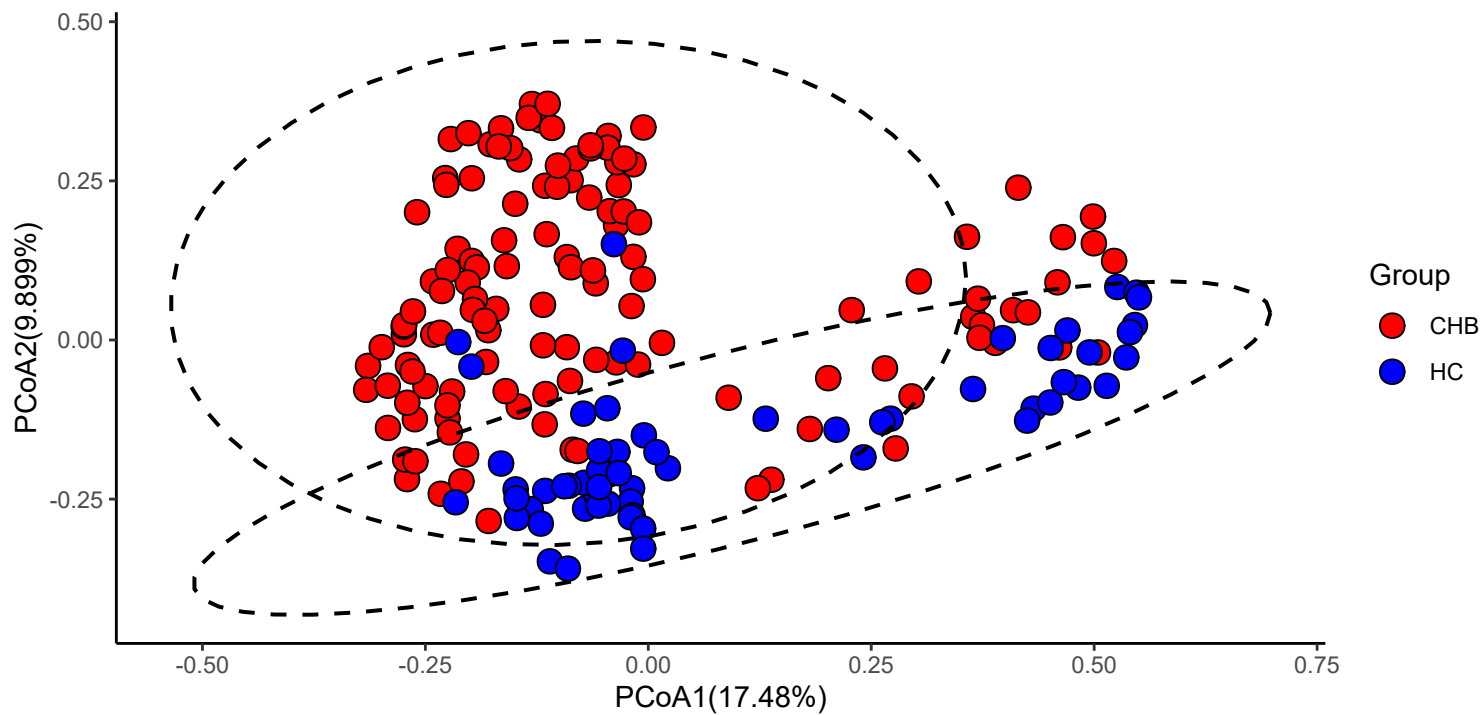

Supplement: Supplementary Figure 1 — Diversity analysis of gut microbiota between CHB and HC. (A) Alpha diversity analysis. CHB patients exhibited reduced gut microbial species richness and diversity compared to HC, as evidenced by significantly lower values of the Chao1 index, Simpson index, and Shannon index. (B) Beta diversity analysis. Principal coordinates analysis (PCoA) based on Bray-Curtis distances revealed significant compositional dissimilarities in gut microbiota between CHB and HC groups (PERMANOVA: R2 = 0.06, P = 0.001). *P < 0.05; **P < 0.01; ***P < 0.001. [file Image_1.pdf]

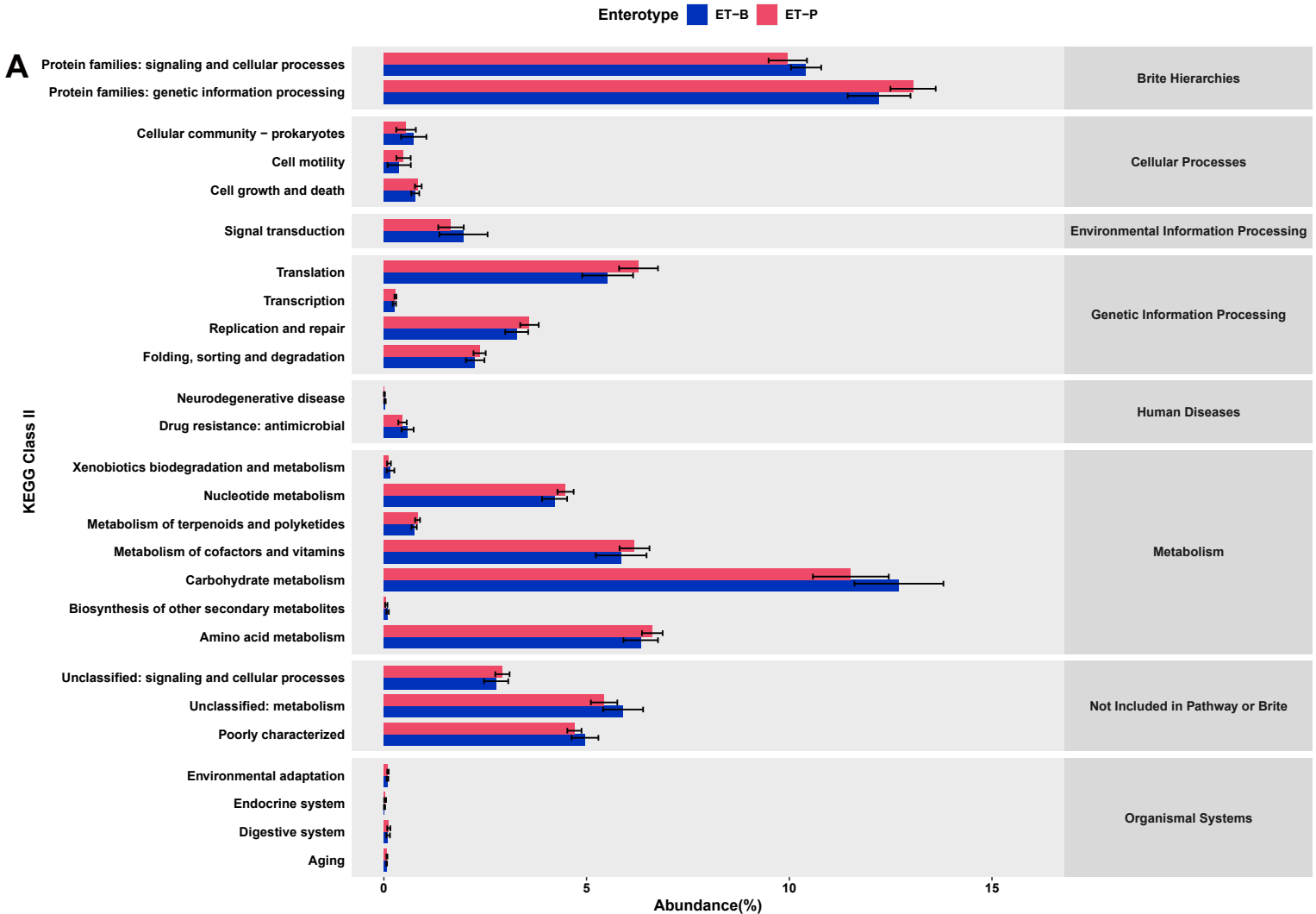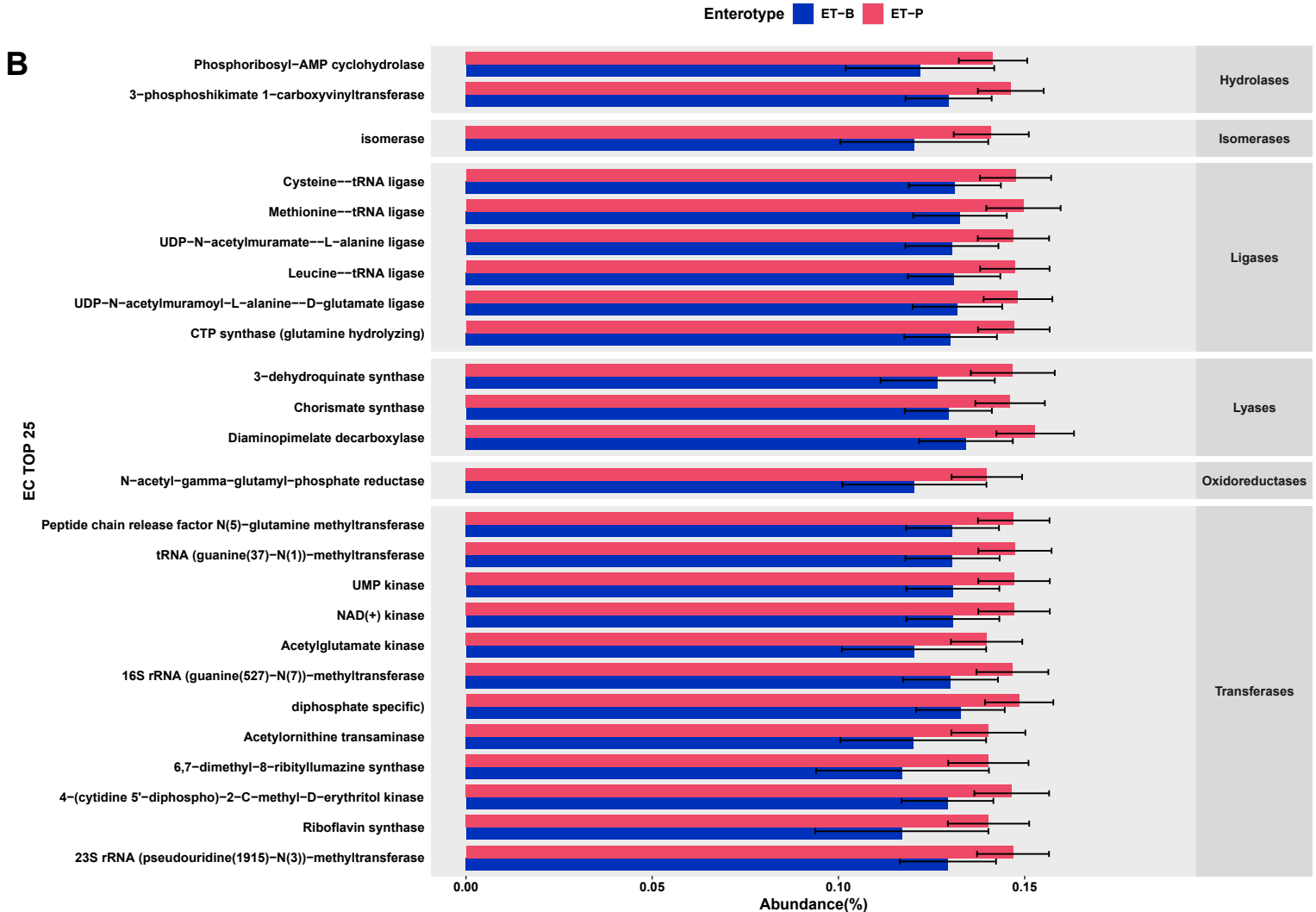

Supplement: Supplementary Figure 2 — Comparative functional annotation of enterotypes based on KEGG Orthology (KO) and Enzyme Commission (EC) profiles. (A) Differentially enriched KEGG pathways between the two enterotypes. Pathways showing significant disparities (threshold: P < 0.05) are ranked by effect size. (B) Top 25 EC categories with the most pronounced differences between enterotypes. [file Image_2.pdf]

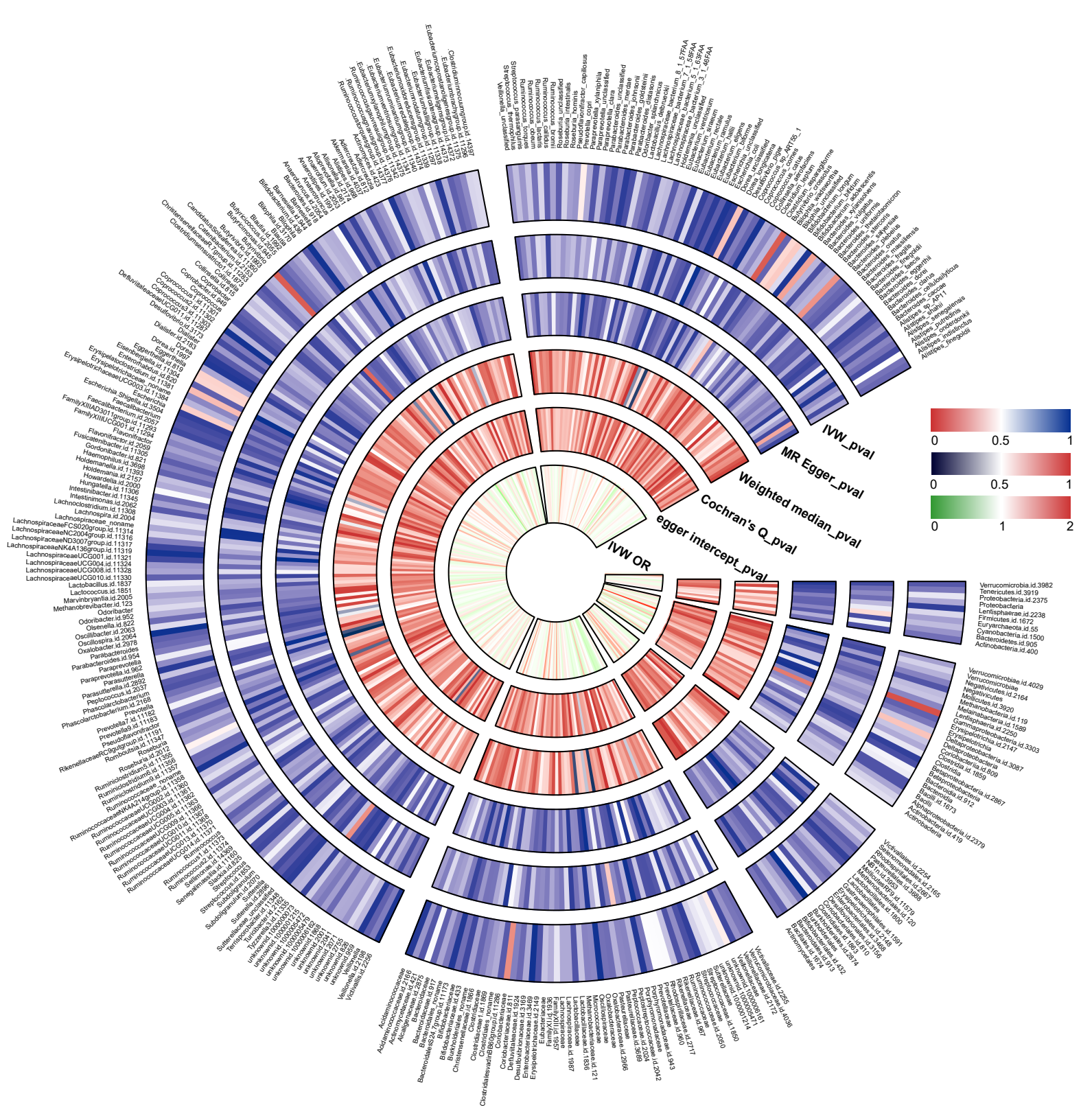

Supplement: Supplementary Figure 3 — The causal link between the taxonomy of 330 gut microbiota and CHB. [file Image_3.pdf]
